# Co-existence of multiple bacterivorous clevelandellid ciliate species in hindgut of wood-feeding cockroaches in light of their prokaryotic consortium

**DOI:** 10.1038/s41598-018-36245-y

**Published:** 2018-12-10

**Authors:** Peter Vďačný, Emese Érseková, Katarína Šoltys, Jaroslav Budiš, Lukáš Pecina, Ivan Rurik

**Affiliations:** 1 Department of Zoology, Comenius University in Bratislava, 842 15 Bratislava, Slovakia; 2 Comenius University Science Park, Comenius University in Bratislava, 841 04 Bratislava, Slovakia; 3 Department of Computer Science, Comenius University in Bratislava, Mlynská dolina F-1, 842 48 Bratislava, Slovakia; 4 Private computer laboratory, 821 07 Bratislava, Slovakia

## Abstract

The hindgut of wood-feeding *Panesthia* cockroaches harbours a diverse microbial community, whose most morphologically prominent members are bacterivorous clevelandellid ciliates. Co-occurrence and correlation patterns of prokaryotes associated with these endosymbiotic ciliates were investigated. Multidimensional scaling based on taxa interaction-adjusted index showed a very clear separation of the hindgut ciliate samples from the ciliate-free hindgut samples. This division was corroborated also by SparCC analysis which revealed strong negative associations between prokaryotic taxa that were relatively more abundant in the ciliate-free hindgut samples and prokaryotic taxa that were more abundant in the ciliate samples. This very likely reflects the grazing behaviour of hindgut ciliates which prefer Proteobacteria, Firmicutes and Actinobacteria, causing their abundances to be increased in the ciliate samples at the expense of abundances of Euryarchaeota and Bacteroidetes which prevail in the hindgut content. Ciliate species do not distinctly differ in the associated prokaryotes, indicating that minute variations in the proportion of associated bacteria might be sufficient to avoid competition between bacterivorous ciliate species and hence enable their co-occurrence in the same host. The nearest free-living relatives of hindgut ciliates have a different pattern of associations with prokaryotes, i.e., alphaproteobacteria are predominantly associated with free-living ciliates while gammaproteobacteria with hindgut ciliates.

## Introduction

The ability to digest wood evolved two times independently in the insect order Blattodea. First in the common ancestor of termites and cryptocercids, and then in the panesthiine cockroaches^1^. Flagellate symbionts play the key role in wood degradation in the phylogenetically lower termites and cryptocercids, while a diverse assemblage of cellulolytic prokaryotes substitutes them in the flagellate-free higher termites^2,3^. The fermentative, hindgut compartment of panesthiine cockroaches also harbours a diverse, anaerobic microbial community. Some of its members are most closely related to prokaryotic lineages from omnivorous cockroaches and wood- or litter-feeding termites, while others have been encountered also in the large intestine of vertebrates^4^. In addition, panesthiines carry in their hindgut morphologically very distinctive ciliates, belonging to three related genera *Clevelandella*, *Paraclevelandia* and *Nyctotherus*^5–9^. These ciliates are, however, not associated with the digestion of wood, as already recognized by Kidder^5^ who explicitly noted that the endoplasm of the hindgut ciliates was filled with food vacuoles containing bacteria and wood was never detected in the vacuoles. Very likely these ciliates contribute to the overall hindgut function by their grazing behaviour, as known for other ciliates living in the gastrointestinal tract^10,11^. Clevelandellid ciliates are strict anaerobes with mitochondria of a hydrogenosome type, which promotes their relationship with hydrogenotrophic methanogens, especially, from the orders Methanobacteriales and Methanomicrobiales^12–15^.

While the genus *Nyctotherus* is species-rich and distributed in various blaberiid and blattid cockroaches^16–19^, *Clevelandella* and *Paraclevelandia* account together for only ten species that have been so far exclusively recorded in the blaberiid genus *Panesthia*. Interestingly, multiple *Clevelandella* and *Paraclevelandia* species might co-occur in a single host and their distribution copies the whole range of their host cockroach genus from Japan to Australia^5–9^. This fact indicates that the common evolutionary ancestor of *Clevelandella* and *Paraclevelandia* was very likely acquired by the progenitor of the genus *Panesthia* or these ciliates were horizontally transferred between co-occurring *Panesthia* species by coprophagy. In this light, panesthiine cockroaches provide an interesting natural laboratory for studying speciation and co-occurrence of several closely related ciliate species.

In the present study, we investigated co-occurrence and correlation patterns of prokaryotes associated with hindgut ciliates from panesthiine cockroaches. We also analyzed whether particular ciliate species differ in the associated archaea and bacteria. To address these issues, we set the following objectives: (1) to describe prokaryotic diversity associated with the hindgut compartment of panesthiine cockroaches and with individual hindgut ciliate species; (2) to construct co-occurrence networks of prokaryotes, using interaction-adjusted indices; and (3) to assess similarity of prokaryotic communities of individual ciliate species.

## Material and Methods

### Material sampling

Two subspecies of *Panesthia angustipennis* were obtained by purchase from commercial breeders. The subspecies *angustipennis* was collected in a tropical rain forest in Khao Yai in the western part of the Sankamphaeng Mountain Range, Nakhon Ratchasima Province, Thailand (14°26′N, 101°22′E). The subspecies *cognatha* was studied in two populations. One came from a tropical rain forest on the Côn Sơn (Grande-Condore) island from the Côn Đảo archipelago of the Bà Rịa–Vũng Tàu Province, in the coastal southeastern Vietnam (8°41′N, 106°34′E). The other one originated from a not closely specified locality in Cambodia. Cockroaches from individual sampling sites were bred separately at room temperature in plastic boxes filled with decaying wood. Specimens were sacrificed in formalin vapours and their hindgut was dissected and placed into a sterile Petri dish containing Ringer’s solution (0.9%). Hindgut content was extracted with a micropipette and observed under an optical microscope Leica DM2500 equipped with differential interference contrast optics. Living ciliates were manually picked from the hindgut content with the aid of specially adjusted Pasteur micropipettes. Isolated ciliates were studied in detail *in vivo* and after protargol impregnation^20^. Identification was based on the study of Kidder^5^ and altogether six distinct morphospecies were recognized: *Clevelandella constricta*, *C*. *hastula*, *C*. *panesthiae*, *C*. *parapanesthiae*, *Paraclevelendia brevis*, and *Nyctotherus* sp.

For molecular analyses, single cells of individual ciliate morphospecies were picked from the hindgut content. They were carefully washed with a modified Pasteur’s micropipette in five drops of Ringer’s solution. In each washing step, micropipettes were changed. Thoroughly washed cells were lyzed in 180 µl of ATL buffer (Qiagen, Hildesheim, Germany) and stored at 6 °C. Each sample contained a single ciliate cell. In total, we obtained 27 ciliate samples (Table 1): four from *C*. *constricta* (three from Thailand and one from Vietnam), 11 from *C*. *hastula* (six from Thailand, three from Vietnam and two from Cambodia), three from *C*. *panesthiae* (all from Vietnam), seven from *C*. *parapanesthiae* (four from Thailand, two from Vietnam and one from Cambodia), one from *P*. *brevis* (Cambodia) and one from *Nyctotherus* sp. (Thailand). The number of samples was proportional to the abundance of individual species in the hindgut, i.e., *C*. *hastula* and *C*. *parapanesthiae* were the most common taxa while *P*. *brevis* and *Nyctotherus* sp. were very rare. Ciliates were not cultivated and were directly picked for molecular analyses. Total DNA was extracted from ciliate samples with the DNeasy^®^ Blood & Tissue Kit (Qiagen, Hildesheim, Germany). This kit is designed also for DNA isolation from bacteria coming from a variety of sample sources and for very small amounts of starting material. To identify the prokaryote community of the hindgut compartment, a volume of 50 µl of the ciliate-free gut content was obtained from each dissected cockroach and mixed with 150 µl of ATL buffer. To maintain the same conditions in all subsequent molecular procedures, DNA was extracted from the hindgut samples with the same kit as from the ciliate samples. To test the quality of DNA isolation from the hindgut samples, a Vietnamese specimen of *P*. *angustipennis cognatha* was randomly chosen and three subsamples were generated: diluted 1:1, 1:3 and 1:5 with sterile distilled water. Again, a volume of 50 µl was taken from each diluted sample and mixed with 150 µl of ATL buffer. Our rationale for dilution was based on assumption that it will further reduce the amounts of potential enzyme inhibitors. Electrophoresis of PCR products obtained from all samples revealed that they were of comparable quality. Moreover, all statistical subsequent analyses showed that the diluted hindgut samples maintained the original signature, corroborating that DNA was successfully isolated also from the undiluted hindgut samples. Characteristics and origin of all samples are summarized in Table 1.

**Table 1:** Characteristics of 16S rRNA gene amplicon libraries of prokaryotic hindgut microbiota of the wood-feeding cockroach *Panesthia angustipennis*.

Sample code	Country of origin	Cockroach subspecies	Material	No. of reads	No. of genus level taxa	Richness^b^	Diversity^b^	Effective number of OTUs^b^
08-Paa-T	Thailand	*angustipennis*	*C*. *constricta*	68 843	145	225.2	3.18	24.10
09-Paa-T	Thailand	*angustipennis*	*C*. *constricta*	104 724	127	151.0	2.66	14.25
10-Paa-T	Thailand	*angustipennis*	*C*. *constricta*	35 092	152	194.7	2.97	19.56
07-Pac-V	Vietnam	*cognatha*	*C*. *constricta*	60 794	144	187.6	3.02	20.49
14-Paa-T	Thailand	*angustipennis*	*C*. *hastula*	46 652	165	203.3	2.95	19.13
15-Paa-T	Thailand	*angustipennis*	*C*. *hastula*	41 215	109	153.1	2.75	15.70
25-Paa-T	Thailand	*angustipennis*	*C*. *hastula*	18 044	112	119.5	2.55	12.79
26-Paa-T	Thailand	*angustipennis*	*C*. *hastula*	23 401	152	195.7	2.57	13.02
29-Paa-T	Thailand	*angustipennis*	*C*. *hastula*	14 635	96	107.1	2.26	9.60
31-Paa-T	Thailand	*angustipennis*	*C*. *hastula*	25 025	105	116.2	2.54	12.68
02-Pac-K	Cambodia	*cognatha*	*C*. *hastula*	58 740	193	243.7	3.21	24.70
03-Pac-K	Cambodia	*cognatha*	*C*. *hastula*	122 333	106	152.7	2.01	7.44
16-Pac-V	Vietnam	*cognatha*	*C*. *hastula*	44 681	156	327.1	3.36	28.65
17-Pac-V	Vietnam	*cognatha*	*C*. *hastula*	60 676	131	149.0	2.70	14.88
21-Pac-V	Vietnam	*cognatha*	*C*. *hastula*	24 440	104	112.0	1.77	5.86
06-Pac-V	Vietnam	*cognatha*	*C*. *panesthiae*	63 853	135	163.2	2.56	12.89
23-Pac-V	Vietnam	*cognatha*	*C*. *panesthiae*	26 908	67	117.0	1.87	6.47
24-Pac-V	Vietnam	*cognatha*	*C*. *panesthiae*	15 199	131	177.7	1.92	6.83
11-Paa-T	Thailand	*angustipennis*	*C*. *parapanesthiae*	30 958	114	151.6	2.77	15.98
27-Paa-T	Thailand	*angustipennis*	*C*. *parapanesthiae*	30 000	104	124.1	2.10	8.13
28-Paa-T	Thailand	*angustipennis*	*C*. *parapanesthiae*	29 194	115	175.1	2.49	12.07
30-Paa-T	Thailand	*angustipennis*	*C*. *parapanesthiae*	27 547	107	114.2	2.17	8.78
04-Pac-K	Cambodia	*cognatha*	*C*. *parapanesthiae*	99 715	97	119.0	2.27	9.63
18-Pac-V	Vietnam	*cognatha*	*C*. *parapanesthiae*	67 333	131	176.6	2.81	16.58
22-Pac-V	Vietnam	*cognatha*	*C*. *parapanesthiae*	38 853	82	90.6	2.29	9.92
19-Pac-K	Cambodia	*cognatha*	*P*. *brevis*	94 038	120	138.0	2.97	19.46
13-Paa-T	Thailand	*angustipennis*	*Nyctotherus* sp.	51 552	152	186.3	3.13	22.75
I20-Paa-T	Thailand	*angustipennis*	Hindgut content	20 487	154	249.6	1.67	5.33
I30-Pac-K	Cambodia	*cognatha*	Hindgut content	14 112	116	142.3	1.45	4.25
I10-Pac-V	Vietnam	*cognatha*	Hindgut content	11 789	157	222.0	2.57	13.00
I11-Pac-V	Vietnam	*cognatha*	Hindgut content^a^	16 257	152	198.0	2.10	8.21
I13-Pac-V	Vietnam	*cognatha*	Hindgut content^a^	14 815	123	175.1	2.32	10.21
I15-Pac-V	Vietnam	*cognatha*	Hindgut content^a^	10 895	149	198.4	3.12	22.60

^a^Three hindgut subsamples were generated from the Vietnamese specimen of *P*. *angustipennis cognatha*: diluted 1:1 (I11-Pac-V), 1:3 (I13-Pac-V) and 1:5 (I11-Pac-V). ^b^All biodiversity indices were based on genus level taxa and were calculated, using the R package SpadeR ver. 0.1.1^64^. Richness, Chao1 estimator^65^; diversity, nonparametric Shannon index^66^; effective number of OTUs, exponential of Shannon entropy^66^.

### PCR amplification, libraries construction and high-throughput sequencing

The fragment of bacterial *16S rDNA* was amplified with the 27f (5′-AGA GTT TGA TCM TGG CTC AG-3′) and the 1492r (5′-CGG TTA CCT TGT TAC GAC TT-3′) primers, while the archaeal *16 rDNA* with the Arc 344 f-mod (5′-ACG GGG YGC ASS AGK CGV GA-3′) and the Arch 958 r-mod (5′-YCC GGC GTT GAV TCC AAT T-3′) primers. Amplification was conducted with the GoTaq Long PCR Master Mix (Promega, Madison, Wisconsin, USA). The thermocycler PCR program consisted of an initial hot start incubation of 15 min at 95 °C followed by 30 identical amplification cycles (denaturing at 95 °C for 45 s, annealing at 55 °C for 1 min, and extension at 72 °C for 2.5 min), and a final extension at 72 °C for 10 min. Quality of amplified DNA was checked by agarose gel electrophoresis and PCR products were consequently purified using the NucleoSpin® Gel and PCR Clean-up system (Macherey-Nagel, Düren, Germany).

The *16S rDNA* full-length amplicons were sequenced using a shot-gun approach. Briefly, the amplicons were digested by transposon-based chemistry and subsequently indexed by low-cycle PCR. Fragments purified by AMPure XP magnetic beads, were quantified fluorometrically using Qubit 2.0 Fluorometer (Invitrogen). Agilent 2100 Bioanalyzer (Agilent Technologies, Waldbronn, Germany) was used for fragment length estimation. The 4 nM final libraries were pooled, diluted to 13 pM and sequenced on Illumina MiSeq platform using v3 chemistry (Illumina, San Diego, CA, USA) and 2 × 300 paired-end run in the Comenius University Science Park (Bratislava, Slovakia).

### Bioinformatics processing of sequencing data

Based on quality control statistics generated by FastQC^21^, adapters and low-quality ends of sequenced reads were removed using Trimmomatic^22^. After trimming, overlapping read pairs were merged with PEAR^23^ and fragments without sufficient length of both reads were removed. Bacterial *16S rRNA gene* sequences were clustered and taxonomies were assigned with the help of the RDP classifier^24^. Archaeal *16S rRNA gene* sequences were processed using the full set of the Silva database^25^ and the Metaxa2 classifier^26^ with a confidence threshold of 80%. Genus level taxa with abundances higher than 0.1% were filtered for the purposes of correlation and similarity analyses.

### Correlation analyses and construction of correlation networks

Interactions between prokaryotic taxa were assessed using the SparCC (Sparse Correlations for Compositional data) technique proposed by Friedman and Alm^27^. This method is suitable for estimating true correlation values from compositional data since it accounts for their specific statistical properties. Standard methods for computing correlations from sequencing data are theoretically invalid because their correlation estimates are biased by the fact that they must sum to 1, fractions are not independent and tend to have a negative correlation regardless of the true correlation between the underlying absolute abundances^27,28^.

Fractions in the co-occurrence matrix were calculated from observed counts in a Bayesian framework. For each sample, the posterior joint fractions were modelled with a Dirichlet distribution, using the package NumPy in Python^29^ and assuming unbiased sampling in the sequencing procedure and a uniform prior. The robustness of downstream analyses and variation of genus level taxa between individual samples were assessed with 100 simulations. The generated posterior joint fractions distribution was log-transformed and true Pearson correlations between components were calculated with the basic SparCC method, as described by Friedman and Alm^27^.

The basic SparCC method was improved by an iterative procedure, including custom Python scripts. To meet a priori SparCC assumptions, that the average covariance is relatively small, pairs with the highest absolute covariance were identified and excluded. In each iteration step, 0.025% of pairs with the highest positive covariance and 0.025% of pairs with the highest negative covariance were removed and covariance was re-calculated using the basic SparCC technique. The whole procedure was repeated while the total covariance significantly decreased during the last 10 iterations. The removal of pairs from the variation matrix followed the iteration technique proposed by Friedman and Alm^27^.

The refined SparCC matrix was used to construct a correlation network, using the scikit-learn^30^ and Matplotlib^31^ packages implemented in Python. Only statistically significant (*p* < 0.05) SparCC correlations, whose absolute value was greater than 0.5, were shown as edges in the network. The node size was indicated by the relative abundance of genus level taxa across the full dataset.

### Interaction-adjusted similarity analyses

The similarity of the analyzed samples was measured by weighted Taxa INteraction-Adjusted (TINA) index proposed by Schmidt *et al*.^32^. According to their statistical analyses, interaction-adjusted indices outperform traditional ones and capture novel aspects of diversity outside the scope of standard approaches. Prior to calculation of TINA index, the SparCC correlation matrix was transformed to a common scale by correlating taxa by their pairwise associations to all other taxa in the matrix and transforming this into a Pearson similarity.

The similarity of our samples, as measured by weighted TINA index, was assessed by metric multi-dimensional scaling implemented in the scikit-learn package in Python^30^. The SMACOF algorithm was run with 200 initializations, each run had 20 000 iterations, and ε was set to 1E–08 to declare convergence. Plotting of the ordination diagram was done with the Matplotlib module^31^. Similarity analyses were based only on bacterial genus level taxa, because archaeal diversity was much smaller, and archaea did not exhibit any distinct distribution pattern among the hindgut and the ciliate samples.

## Results and Discussion

A complex microbial community was revealed in the hindgut of the wood-feeding cockroach *Panesthia angustipennis* (Blattodea: Blaberidae). Its morphologically most conspicuous members were represented by ciliates from three genera of the order Clevelandellida (Ciliophora: Armophorea): *Clevelandella* and *Paraclevelandia* from the family Clevelandellidae, and *Nyctotherus* from the family Nyctotheridae. Altogether six species were recognized: *C*. *constricta*, *C*. *hastula*, *C*. *panesthiae*, *C*. *parapanesthiae*, *P*. *brevis* and *Nyctotherus* sp. Their main morphological characters were summarized in Supplementary Table S1. These ciliates graze prokaryotes with the aid of a conspicuous zone of membranelles that extends through the vestibulum towards the cytostome. Members of all three ciliate genera have been recorded in various *Panesthia* species from Japan to Australia^5–9^, indicating that they represent a stable component of the panesthiine hindgut microbiota.

### Structure of the archaeal community

**Figure 1:**
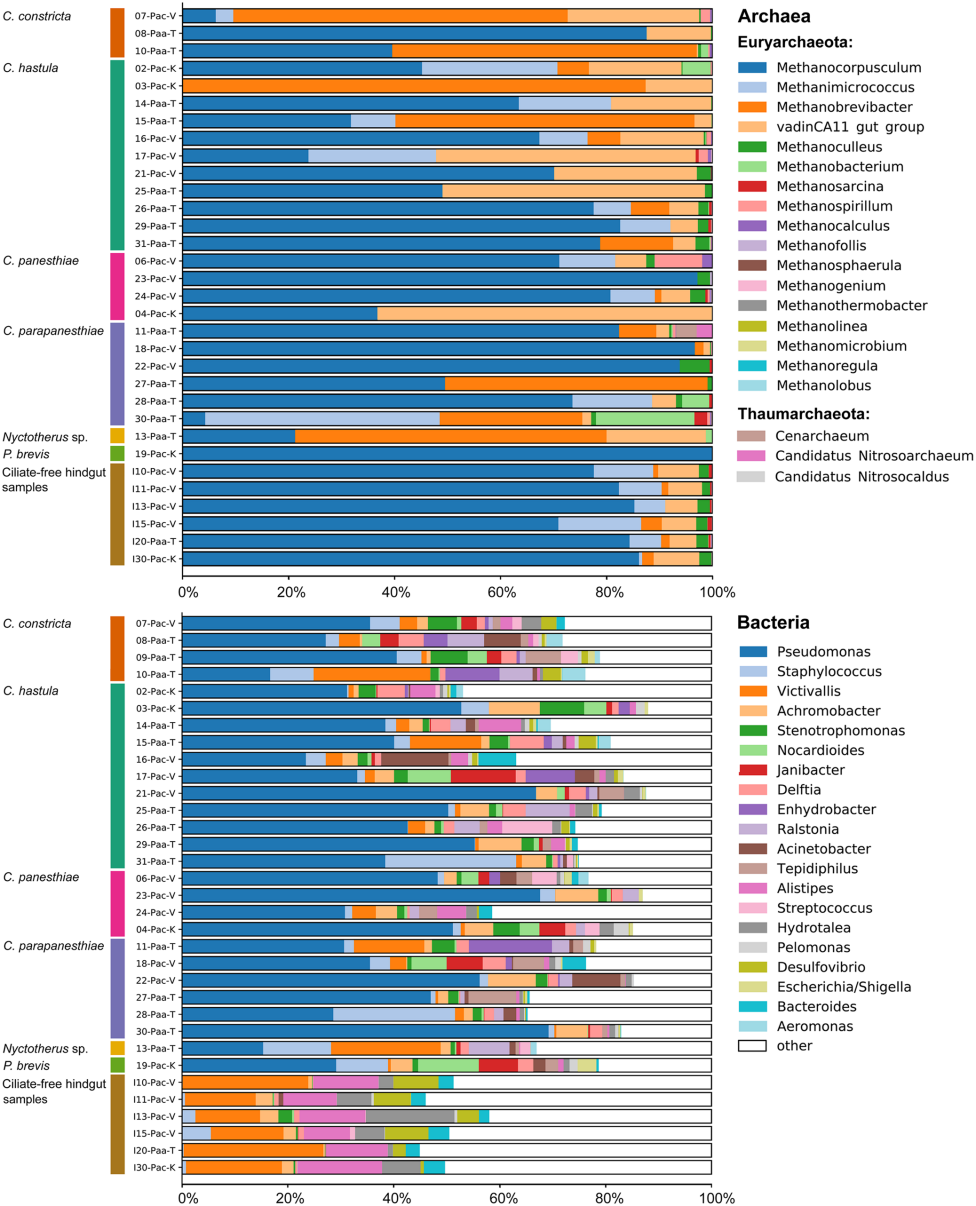
Bar graphs for relative abundances of most common genus level taxa from the domain Archaea (upper panel) and the domain Bacteria (lower panel) in hindgut ciliate samples and ciliate-free hindgut samples collected from *Panesthia angustipennis*. For sample codes, see Table 1.

**Figure 2:**
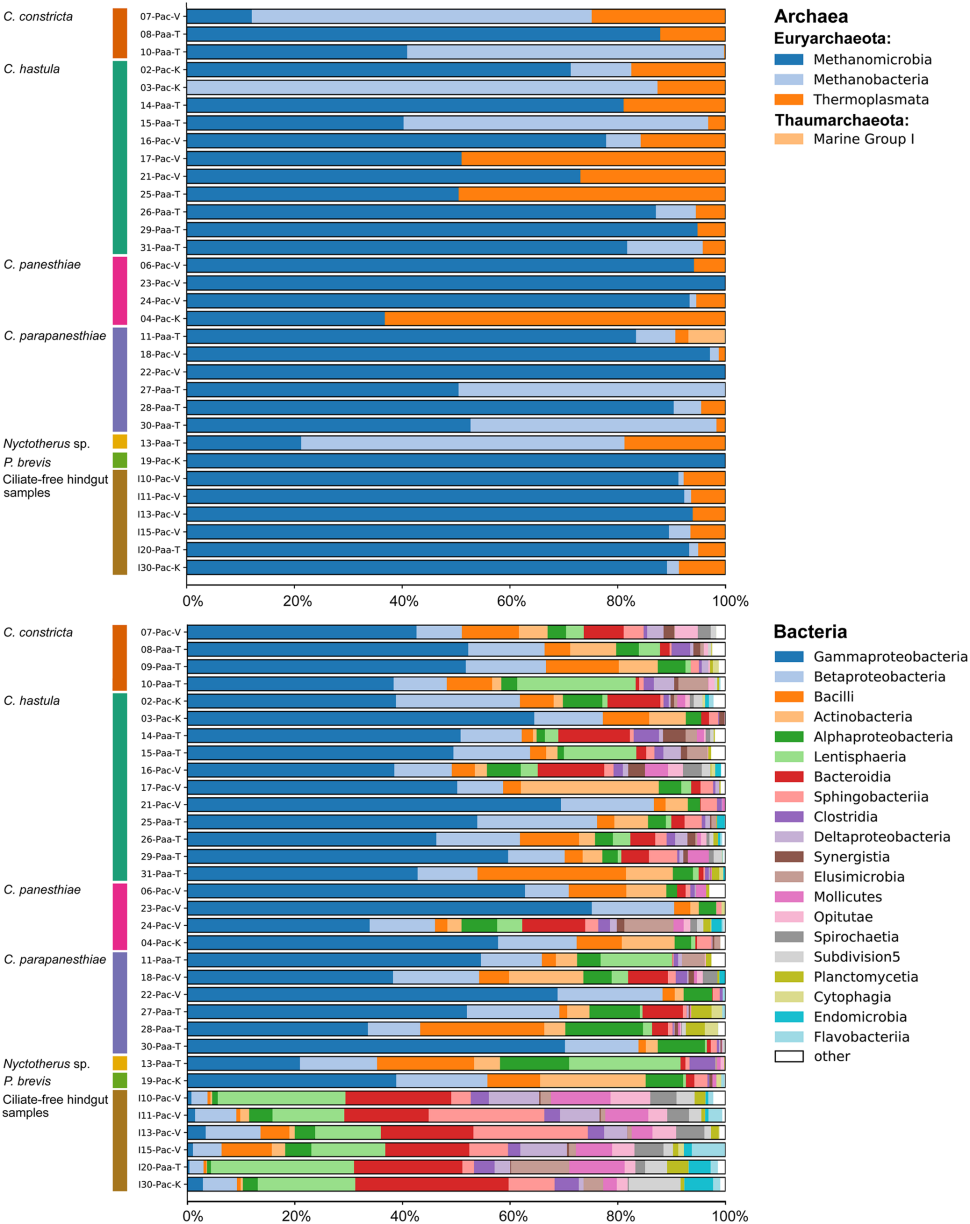
Bar graphs for relative abundances of classes from the domain Archaea (upper panel) and the domain Bacteria (lower panel) in hindgut ciliate samples and ciliate-free hindgut samples collected from *Panesthia angustipennis*. For sample codes, see Table 1.

After quality filtering, altogether 154 326 reads of archaea were retained. The archaeal community of *P*. *angustipennis* was comparatively poor, with predominance of methanogens. All archaeal reads were classified in only 24 genus level taxa belonging to two groups: the under-represented Thaumarchaeota and the over-dominated Euryarchaeota (Figs 1 and 2, upper panels). All Euryarchaeota reads were assigned to only four orders: Methanobacteriales, Methanomicrobiales, Methanosarcinales, and Thermoplasmatales. Relative abundances were, however, very unevenly distributed within them and usually only a single genus distinctly prevailed in each order (Supplementary Table S2). Thus, in the ciliate-free hindgut samples, the most abundant (average relative abundance ± SD between samples) were *Methanocorpusculum* (0.81 ± 0.053) from the Methanomicrobiales, *Methanimicrococcus* (0.08 ± 0.047) from the Methanosarcinales, and the vadinCA11 gut group (0.07 ± 0.011) from the Thermoplasmatales. It is noteworthy that Hara *et al*.^33^ also recognized that the Methanosarcinales and lineages related to the Thermoplasmatales are important components of the archaeal gut communities in *P*. *angustipennis*. Thermoplasmatales are now referred to as Methanomassiliicoccales^34^ and represent the seventh order of methanogens with a unique, obligately hydrogen-dependent methylotrophic metabolism^35^. The few detected hindgut Thaumarchaeota reads were assigned to the Marine Group I by the Metaxa2 classifier.

In the hindgut ciliate samples, the most abundant were *Methanocorpusculum* (0.59 ± 0.300), members of the vadinCA11 gut group (0.13 ± 0.167), and *Methanobrevibacter* (0.17 ± 0.260) from the Methanobacteriales. It is well known that the cytoplasm of *Nyctotherus* is densely colonized by *Methanobrevibacter* species^36,37^. *Methanobrevibacter* reads were, however, detected not only in the *Nyctotherus* sample but also in the *Clevelandella* and *Paraclevelandia* samples as well as in the ciliate-free hindgut samples, indicating their acquisition from the hindgut content. Phylogenetic analyses of van Hoek *et al*.^37^ strongly suggest multiple acquisitions of methanogenic archaea from environmental sources in various ciliate groups. Since *Nyctotherus*, *Clevelandella* and *Paraclevelandia* are closely related^5,9,38,39^, a vertical transmission of methanogenic endosymbionts cannot be also excluded. Nevertheless, it remains to be investigated whether traces of host phylogeny can be found in the archaeal microbiota in the guts of cockroaches. It is also important to mention that methanogens are not present only in wood-feeding cockroaches but possibly in all dictyopteran lineages^18,40,41^.

### Structure of the bacterial community

In total, 1 363 371 high-quality sequence reads were obtained from *P*. *angustipennis* and they were assigned to 270 genera, when the >0.1% abundance filter was applied. This value is comparatively like those provided by Dietrich *et al*.^42^ for two related wood-feeding cockroaches, i.e., they reported 202 genus level taxa from *P*. *angustipennis* and 296 from *Salganea esakii*. As already recognized by Bauer *et al*.^4^, some bacteria from panesthiine cockroaches are typical members of gut assemblages in omnivorous cockroaches and wood- or litter-feeding termites (e.g., *Desulfovibrio*, *Dysgonomonas*, *Rikenella* and *Ruminococcus*), while others have been encountered in the large intestine of vertebrates (e.g., *Victivallis* and subdivision 5 genera). This supports the selection hypothesis that the structure of the intestinal community of the wood-feeding panesthiines is shaped by a selection of lineages with ecological amplitude that matches the conditions in the respective microenvironments.

The phylum Bacteroidetes clearly predominated (0.32 ± 0.068) in the hindgut samples (Supplementary Table S3), which is in accordance with Dietrich *et al*.^42^ and Bauer *et al*.^4^. The other abundant phyla were (arranged in decreasing order; Supplementary Table S3): Lentisphaerae (0.18 ± 0.055), Proteobacteria (0.16 ± 0.053), Verrucomicrobia (0.08 ± 0.026), Tenericutes (0.07 ± 0.031), and Firmicutes (0.06 ± 0.029). The most abundant genus level taxa in the *P*. *angustipennis* hindguts were (Supplementary Table S4): *Victivallis* (0.18 ± 0.054) from the phylum Lentisphaerae as well as *Alistipes* (0.12 ± 0.022) and *Hydrotalea* (0.07 ± 0.05) from the phylum Bacteroidetes (Fig. 1, lower panel). *Alistipes* was also recognized as a highly abundant bacterium in the panesthiine cockroaches by Bauer *et al*.^4^. Interestingly, members of the genus *Alistipes* are known to be proteolytic^43^, which mirrors well the protein-rich diet (decaying wood infested with fungi) of wood-feeding cockroaches, as already suggested by Bauer *et al*.^4^. Such selection of bacterial lineages with similar functions by specific environmental conditions is also corroborated by the comparatively high relative abundance of *Hydrotalea* (0.07 ± 0.05) from the family Chitinophagaceae, whose members are capable of degrading chitinous fungal cell walls. Proteobacteria were represented in the panesthiine hindgut mostly by the sulfate-reducing *Desulfovibrio* (0.05 ± 0.03) from the class Deltaproteobacteria. Interestingly, Verrucomicrobia subdivision 5 genera were only slightly less abundant (0.04 ± 0.028) than *Desulfovibrio*. Members of subdivision 5 have been commonly recorded also on mucous layers in the large intestine of various animals, where they could forage on sulfated glycopolymers produced in such habitats^44^. As typical for omnivorous and wood-feeding cockroaches, Elusimicrobia (0.05 ± 0.051) and Spirochaetes (0.04 ± 0.019) were rare. In our samples, they were represented by the genus *Treponema* (0.03 ± 0.019) from the phylum Spirochaetes, and by the genus *Elusimicrobium* (0.03 ± 0.035) and “*Candidatus* Endomicrobium” (0.02 ± 0.019) from the phylum Elusimicrobia (Supplementary Table S4). Their occurrence in the ciliate-free hindgut samples supports the presence of free-living relatives of ectosymbiotic spirochaetes and endosymbiotic endomicrobia in cockroaches and termites, before they were recruited as symbionts of flagellates in lower termites^42,45,46^.

Ciliate samples strongly differed in the bacterial community structure from the ciliate-free hindgut samples already at the phylum level. Specifically, the relative abundances of Proteobacteria (0.70 ± 0.125) distinctly prevailed over those of Bacteroidetes (0.06 ± 0.045) and Lentisphaerae (0.04 ± 0.06) in the ciliate samples. The second most abundant phylum in the ciliate samples was Firmicutes, whose relative proportion (0.09 ± 0.067) was only slightly higher than that of Bacteroidetes, however. The genus *Pseudomonas* (0.41 ± 0.143) from the class Gammaproteobacteria clearly over-dominated in all ciliate samples (Fig. 1, lower panel). It was followed by *Staphylococcus* from the phylum Firmicutes, whose relative abundance was already by one order of magnitude lower (0.05 ± 0.062). This pattern is, however, quite different from that observed in metopids (Armophorea: Metopida) which are the nearest free-living relatives of the hindgut clevelandellids^9,38,39^. Metopids are predominantly associated with Alphaproteobacteria (~85%) which are followed by Gammaproteobacteria (~7.5%)^47^, similarly as it is in other free-living protists^48–50^. The different pattern of associations between endosymbiotic ciliates and bacteria (Fig. 2, lower panel) is thus very likely a result of selection response to the conditions in the microenvironment of the panesthiine hindgut. The genus *Pseudomonas*, which was most commonly associated with the clevelandellids in our samples, is known to contain the secretion system type VI (T6SS). This system might facilitate the survival of the intracellular bacterium and its communication with the eukaryotic host^50,51^. The predominance of *Pseudomonas* in ciliate samples indicates that the secretion system might be of high significance in bacteria-ciliate associations in the hindgut of the panesthiine cockroaches.

### Similarity of samples and prokaryote associations

**Figure 3:**
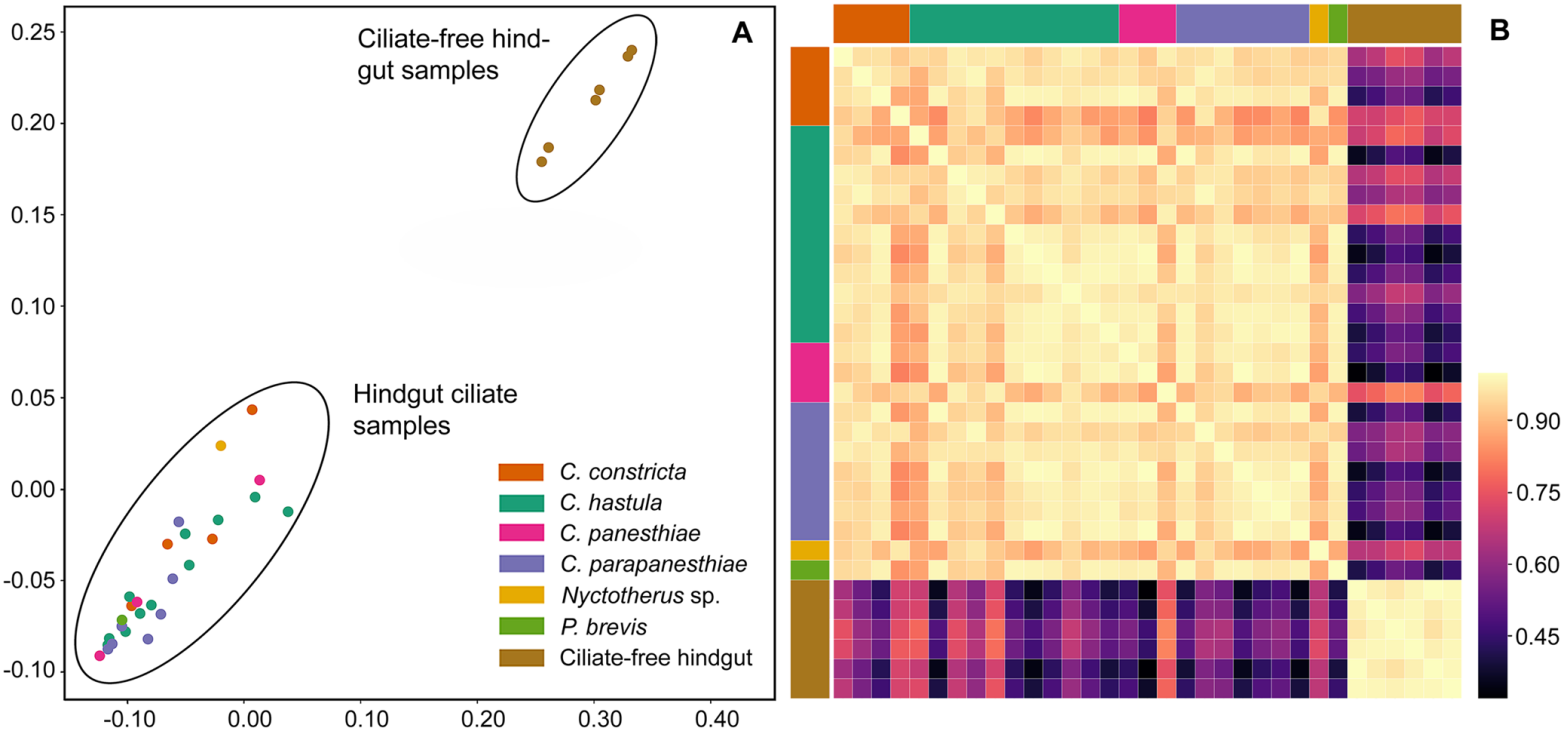
Metric multidimensional scaling plot (**A**) and heat map (**B**) of 32 samples collected from the *Panesthia angustipennis* hindgut. Similarity was measured with TINA index. The ciliate-free gut content samples form a small compact cluster that is distinctly separated from the hindgut ciliate samples.

**Figure 4:**
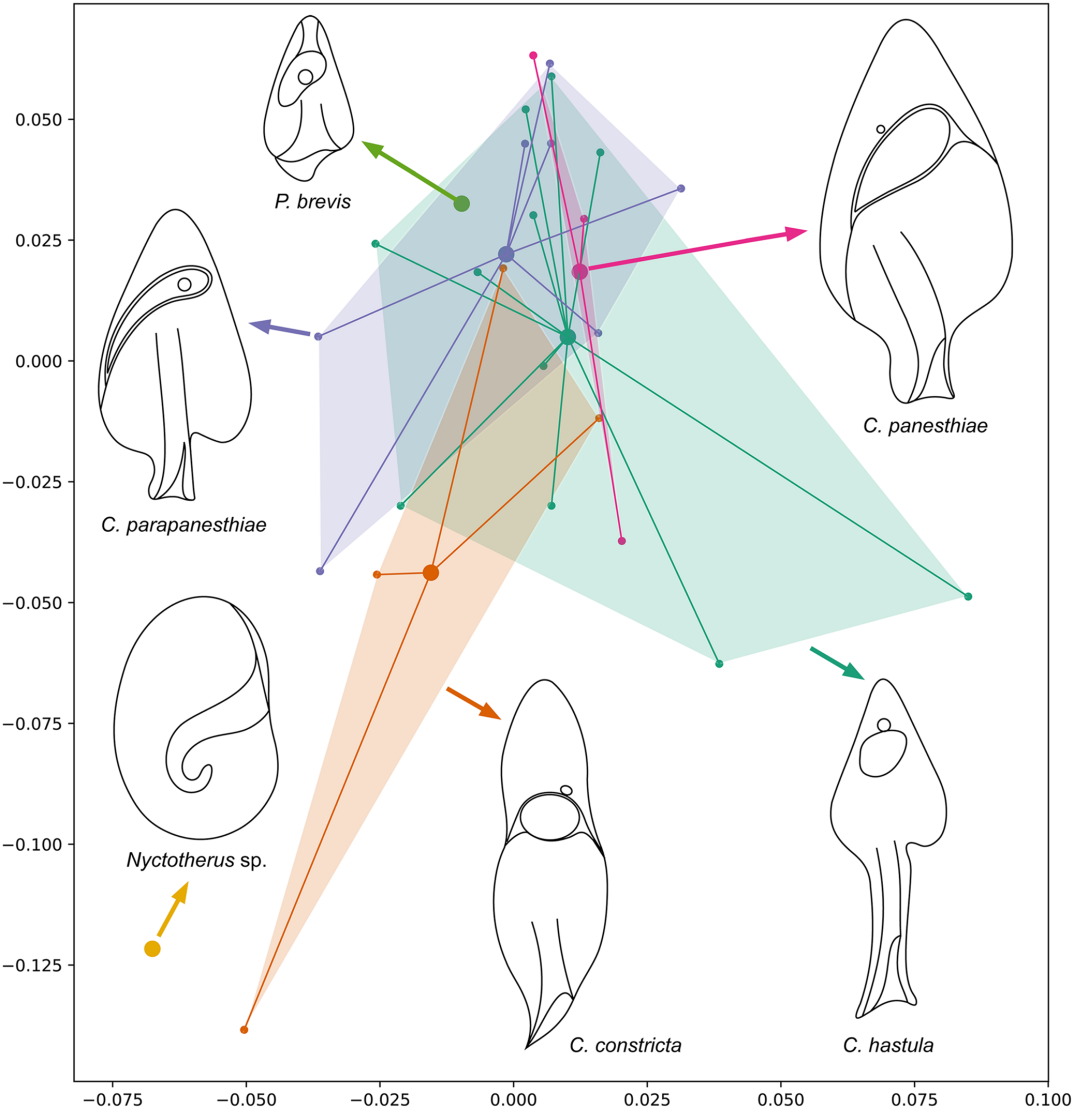
Metric multidimensional scaling plot of 26 samples obtained from six ciliate species isolated from the *Panesthia angustipennis* hindgut. Similarity of samples was measured with TINA index. Samples obtained from the same ciliate species are not grouped together, indicating that the interindividual variability in associated bacteria with ciliates is larger than the intraspecies one.

Multidimensional scaling and heat maps based on weighted TINA index showed a very clear separation of the ciliate-free hindgut samples and the hindgut ciliate samples (Fig. 3). The ciliate-free hindgut samples formed a small compact cluster without any differentiation, which indicates that bacterial communities of even geographically distant populations of *P*. *angustipennis* are similar at the genus level of bacteria. Moreover, after dilution of the hindgut content, the samples maintain the original signature and are still clearly distinct from the hindgut ciliate samples. Likewise, the ciliate samples formed a homogenous but large cluster. However, samples obtained from the same ciliate species were not grouped together in distinct subclusters (Fig. 4), documenting that the interindividual variability of ciliate species in the associated bacterial genus level taxa is larger than intraspecies variability. Also, bar graphs show that the amplitude of interindividual variability is comparatively large (Fig. 1, lower panel). Such a distribution pattern might be a result how individual ciliates avoid competition, which in turn enables co-occurrence of multiple hindgut ciliates in the same host.

**Figure 5:**
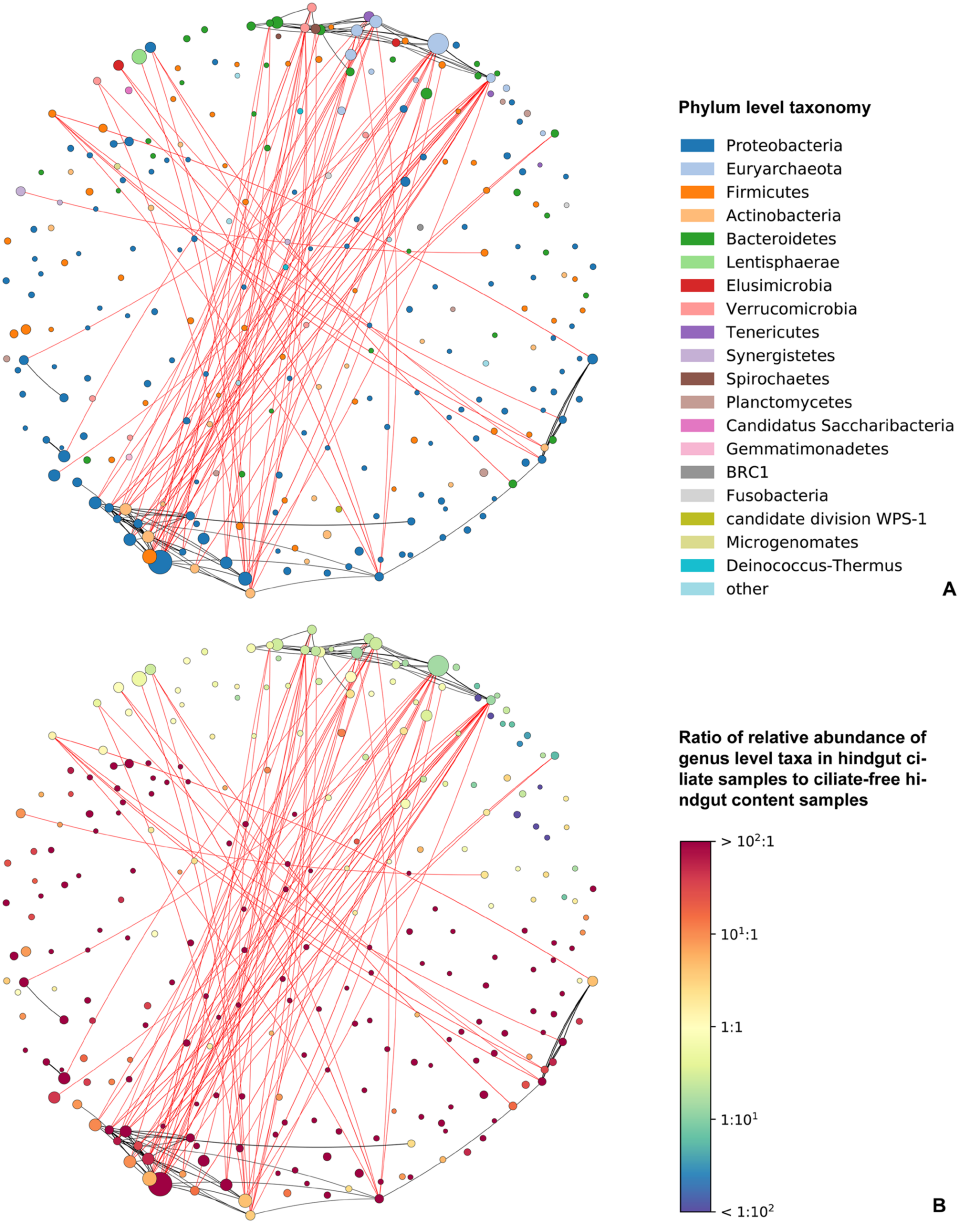
Taxa co-occurrence network based on 32 samples collected from the *Panesthia angustipennis* hindgut, comprising a total of 270 genus level taxa whose abundances were higher than 0.1%. Pairwise SparCC correlations, whose absolute value was greater than 0.5 (*p* < 0.05), are shown as edges in the network. Node size indicates the relative abundance of genus level taxa across the full dataset. Node colour reflects the phylum level taxonomy in panel (A) and the ratio of relative abundance of genus level taxa in the hindgut ciliate samples to the ciliate-free hindgut content samples in panel (B).

After applying the SparCC technique, we recognized only 159 statistically significant associations with absolute value of correlation coefficients greater than 0.5, corroborating the supposition that relative abundances of most taxa are not correlated with each other^27^. Correlation network analyses clearly divided genus level taxa into two groups, separated by a distinct bunch of negative correlations (Fig. 5, red curved lines). The first group contained mostly taxa whose proportion was higher in the ciliate-free hindgut content, while the second group united taxa having a higher affinity to the hindgut ciliate samples. Members of each group were interconnected by a set of positive correlations.

In the first group, a number of positive associations was revealed, especially, between members of the Euryarchaeota, Bacteroidetes, Spirochaetes, and Verrucomicrobia. As concerns archaea, the strongest correlations were found between *Methanocorpusculum* and *Methanoculleus* (*r* = 0.77, *p* < 0.001), *Methanimicrococcus* and the vadinCA11 gut group (*r* = 0.69, *p* < 0.001), *Methanimicrococcus* and *Methanoculleus* (*r* = 0.63, *p* < 0.001) as well as between *Methanocorpusculum* and the vadinCA11 gut group (*r* = 0.62, *p* < 0.001). This indicates that related taxa very likely inhabit similar niches, but do not dominate by competitive exclusion^52^. Also, Friedman and Alm^27^ as well as Schmidt *et al*.^32^ observed a higher likelihood of positive correlations between phylogenetically related taxa, when the SparCC technique was employed. As concerns Spirochaetes, they were mostly positively associated with Bacteroidetes, e.g., *Treponema* with *Paludibacter* (*r* = 0.79, *p* < 0.001) or with *Bacteroides* (*r* = 0.64, *p* < 0.001). Interestingly, relative abundances of Verrucomicrobia subdivision 5 genera were positively correlated with those of archaea (e.g., with *Methanimicrococcus*, *r* = 0.62, *p* < 0.001) and spirochaetes (e.g., with *Treponema*, *r* = 0.62, *p* < 0.001).

By contrast, in the second group, positive relationships between members of Actinobacteria, Alpha-, Beta- and Gammaproteobacteria dominated. The strongest positive correlations were revealed between the actinobacteria *Janibacter* and *Nocardioides* (*r* = 0.80, *p* < 0.001), the alphaproteobacteria *Caulobacter* and *Phenylobacterium* (*r* = 0.97, *p* < 0.001), the betaproteobacterium *Burkholderia* and the actinobacterium *Microbacterium* (*r* = 0.79, *p* < 0.001) as well as between the gammaproteobacteria *Enhydrobacter* and *Moraxella* (*r* = 0.86, *p* < 0.001). However, these association patterns may only reflect that the hindgut ciliates predominantly graze Actinobacteria, Alpha-, Beta- and Gammaproteobacteria, which would cause their relative abundances to be concomitantly increased in the ciliate samples. But we also cannot exclude that some true positive associations have evolved.

Strong negative associations were revealed between members of the first intestinal and the second ciliate sample group, involving Euryarchaeota and Bacteroidetes one hand and Proteobacteria, Firmicutes and Actinobacteria on the other one. The strongest negative correlations were encountered between the bacteroidete *Hydrobacter* and the gammaproteobacterium *Pseudomonas* (*r* = −0.83, *p* < 0.001) as well as between the archaeon *Methanoculleus* and the firmicute *Staphylococcus* (*r* = −0.71, *p* < 0.001), *Pseudomonas* (*r* = −0.70, *p* < 0.001) and the actinobacterium *Nocardioides* (*r* = −0.70, *p* < 0.001). As expected, members of the first groups were relatively more abundant in the ciliate-free hindgut samples, while taxa of the second group in the ciliate samples. This fact may reflect the grazing behaviour of the hindgut ciliates that prefer Proteobacteria, Firmicutes and Actinobacteria.

It is noteworthy that a structured multiple endosymbiosis of archaea and bacteria might have evolved in the hindgut and/or in the hindgut ciliates. Edgcomb *et al*.^53^ suggested that sulfate reducers, methanogens, and anaerobic ciliates might elegantly and intricately meld the sulfur cycle with cycling of carbon and nitrogen. In their proposal, lactate available from ciliate fermentation processes could be used for the growth of the sulfate-reducing bacteria. Methanogens could utilize the hydrogen produced by the ciliate’s hydrogenosomes along with CO_2_ diffused from the ciliate’s metabolism to produce methane. This methane could possibly be oxidized anaerobically via the coupling of this oxidation with the reduction of sulfate by sulfate-reducing bacteria^54,55^. Indeed, we have detected positive correlations between the sulfate-reducing *Desulfovibrio* and the methanogens *Methanimicrococcus* (*r* = 0.30, *p* = 0.086) and the vadinCA11 gut group (*r* = 0.38, *p* = 0.029). Since these associations are only weak and/or statistically insignificant, they are very likely not prevalent in the panesthiine cockroaches. Dietary habits and evolutionary constraints of the hindgut, including the anatomical structure, suitability of the epithelium for microbial attachment and distribution of anoxic conditions, very likely govern the diversity, abundance and metabolic activities of methanogenic archaea^40,56^ and hence also their putative associations with ciliates and sulfate-reducing bacteria. It remains to be investigated whether traces of the supposed structured multiple symbiosis can be found in the microbiota of the panesthiine cockroaches.

### Diversification of hindgut ciliates in the light of associated prokaryotes

Two modes might have shaped diversification of the hindgut ciliates: sympatric speciation within the same host in the absence of geographical isolation or allopatric speciation with geographical isolation of host populations. Both diversification models have been also suggested to occur in free-living and symbiotic ciliates^57–59^. While mechanisms of allopatric speciation are well understood, those driving sympatric speciation are still contentious topics (for a review see, Coyne and Orr^60^). Commonly proposed mechanisms of sympatric speciation include host shifts, ecological isolation within the host, and spontaneous changes in mating selection due to genomic alterations^61^.

*Clevelandella* and *Paraclevelandia* species have been so far exclusively recorded in *Panesthia*, and their distribution copies the whole geographic range of their host genus from Japan and China through India, islands of Indonesia, Melanesia and Micronesia to Australia^5–9,62^. The literature and our data show that clevelandellid species live in sympatry in a huge geographic area at the recent time. Tubay *et al*.^63^ demonstrated that coexistence of even many species becomes possible by building a simple lattice Lotka-Volterra model of competition with minute differences in suitable microhabitats. We also observed only a small average variation in the proportion of dominant bacterial genera associated with clevelandellid species. However, these subtle proportion differences may be sufficient to avoid competition between individual ciliates. Since the six studied clevelandellid species co-occur and do not distinctly differ in the associated bacteria and archaea, they could also have evolved in sympatry by involving disruptive natural selection along with minute variations in species-specific associations and/or by genetically driven reproductive isolation.

Another explanation of the diversity patterns observed is the allopatric speciation model with secondary contact of hindgut ciliate species by the means of horizontal transfer via coprophagy of co-occurring *Panesthia* species. Indeed, our and recent studies indicate that microbial lineages in different host groups of cockroaches could have been acquired independently from the environment^4,42^. Moreover, Bauer *et al*.^4^ argued that at least in wood-feeding cockroaches, selection by the host environment plays a more important role in shaping the intestinal communities than vertical transmission of particular microbial lineages. This selection hypothesis makes the allopatric speciation model with secondary contact by horizontal transfer also likely. Finally, we cannot also rule out that some clevelandellid species evolved in sympatry, while others in allopatry and later have become sympatric.

## Conclusion

A diverse microbial community is developed in the hindgut compartment of wood-feeding cockroaches of the genus *Panesthia*. Relative abundances of Proteobacteria, Firmicutes, and Actinobacteria are increased in the ciliate samples at the expense of relative abundances of Euryarchaeota and Bacteroidetes, which most likely reflects the grazing behaviour of the hindgut ciliates. *Pseudomonas* was the most abundant prokaryote associated with the hindgut ciliates, which indicates a high significance of the secretion system type VI for survival and communication of this gammaproteobacterium with the endosymbiotic hindgut ciliates. Ciliate species do not distinctly differ in the associated prokaryotes, indicating that minute variations in the proportion of associated bacteria might be sufficient to avoid competition between bacterivorous ciliate species and hence enable their co-occurrence in the same host. The nearest free-living relatives of the hindgut ciliates have a different pattern of associations with prokaryotes, i.e., alphaproteobacteria are predominantly associated with free-living ciliates while gammaproteobacteria with hindgut endosymbiotic ciliates. This is very likely a result of selection response to the conditions in the microenvironment of the panesthiine hindgut.

## Electronic supplementary material

Supplementary information

## Data Availability

Results of all analyses are included in this published article and its Supplementary information files. The datasets generated and/or analysed during the current study are available from the corresponding author on reasonable request.
