# Supplementary material for: Co-existence of multiple bacterivorous clevelandellid ciliate species in hindgut of wood-feeding cockroaches in light of their prokaryotic consortium
Source: Sci Rep. 2018 Dec 10;8:17749. doi: 10.1038/s41598-018-36245-y (PMC6288088; doi:10.1038/s41598-018-36245-y)
Supplement: Supplementary file 1 — Supplementary information [file 41598_2018_36245_MOESM1_ESM.doc]

**Supplementary material**

**Co-existence of multiple bacterivorous clevelandellid ciliate species in hindgut of wood-feeding cockroaches in light of their prokaryotic consortium**

**Peter Vďačný^1^, Emese Érseková^1^, Katarína Šoltys^2^, Jaroslav Budiš^3^, Lukáš Pecina^1^ & Ivan Rurik^4^**

^1^Department of Zoology, Comenius University in Bratislava, 842 15 Bratislava, Slovakia. ^2^Comenius University Science Park, Comenius University in Bratislava, 841 04 Bratislava, Slovakia. ^3^Department of Computer Science, Comenius University in Bratislava, Mlynská dolina F-1, 842 48 Bratislava, Slovakia. ^4^Private computer laboratory, 821 07 Bratislava, Slovakia. Correspondence and requests for materials should be addressed to P.V. (email: [peter.vdacny@uniba.sk](mailto:peter.vdacny@uniba.sk))

**Pages: 5**

**Tables: 4**

**Supplementary Table S1.** Characterization of endosymbiotic ciliates isolated from the hindgut of the wood-feeding cockroach *Panesthia angustipennis*.

| **Characteristic** | ***Nyctotherus* sp.** | ***C. constricta*** | ***C. hastula*** | ***C. panesthiae*** | ***C. parapanesthiae*** | ***P. brevis*** |
| --- | --- | --- | --- | --- | --- | --- |
| Body size^a^ | 45‒70 × 30‒50 µm | 80‒150 × 20‒45 µm | 65‒90 × 20‒30 µm | 85‒110 × 35‒55 µm | 60‒95 × 30‒55 µm | 30‒50 × 15‒30 µm |
| Body shape | Ovoid | Cylindrical, constricted at level of macronucleus | Lanceolate with left margin slightly notched and recurved towards peristomal projection | Lanceolate | Lanceolate with left margin distinctly notched and recurved towards peristomal projection | Lanceolate |
| Peristomal projection | Absent | Inconspicuous | Long, occupies 1/2 of body length | Short, occupies 1/6 of body length | Short, occupies 1/6 of body length | Absent |
| Macronucleus | Ovoid, anterior portion surrounded by dark granules, long axis parallel to transverse body axis | Ovoid, long axis parallel to transverse body axis | Ovoid, long axis slightly oblique to main body axis | Cuneate, anterior end rounded and posterior end curved towards right body margin | Narrowly cuneate, anterior end rounded and posterior end curved towards right body margin | Ovoid, long axis slightly oblique to main body axis |
| Karyophore | Present, attached to right and left body margin | Present, attached to right and left body margin | Absent | Present, attached to right and left body margin | Present, attached to only right body margin | Present, attached to anterior body end |
| Adoral membranelles, number^a^ | 46‒65 | 37‒60 | 30‒35 | 43‒50 | 30‒40 | 14‒20 |
| Type host^b^ | *P. angustipennis angustipennis* | *P. javanica* | *P. javanica* | *P. javanica* | *P. javanica* | *P. javanica* |

^a^ Data based on randomly selected, protargol-impregnated specimens (*n* = 20 for each *Clevelandella* species and *n* = 10 for *Nyctotherus* sp. and *P. brevis*).

^b^ *P. javanica* is now considered as a junior synonym of *P. angustipennis angustipennis*.

**Supplementary Table S2.** Relative abundances (in %) of archaeal genera in the samples studied.

| **Genus** | **Order** | **Class** | **Phylum** | **M_tot_** | **M_Cil_** | **M_Int_** | **07-Pac-V** | **08-Paa-T** | **10-Paa-T** | **02-Pac-K** | **03-Pac-K** | **14-Paa-T** | **15-Paa-T** | **16-Pac-V** | **17-Pac-V** | **21-Pac-V** | **25-Paa-T** | **26-Paa-T** | **29-Paa-T** | **31-Paa-T** | **06-Pac-V** | **23-Pac-V** | **24-Pac-V** | **04-Pac-K** | **11-Paa-T** | **18-Pac-V** | **22-Pac-V** | **27-Paa-T** | **28-Paa-T** | **30-Paa-T** | **13-Paa-T** | **19-Pac-K** | **I10-Pac-V** | **I11-Pac-V** | **I13-Pac-V** | **I15-Pac-V** | **I20-Paa-T** | **I30-Pac-K** |
| --- | --- | --- | --- | --- | --- | --- | --- | --- | --- | --- | --- | --- | --- | --- | --- | --- | --- | --- | --- | --- | --- | --- | --- | --- | --- | --- | --- | --- | --- | --- | --- | --- | --- | --- | --- | --- | --- | --- |
| *Methanocorpusculum* | Methanomicrobiales | Methanomicrobia | Euryarchaeota | 63.02 | 58.85 | 81.09 | 6.27 | 87.56 | 39.57 | 45.20 | 0.00 | 63.46 | 31.79 | 67.34 | 23.77 | 70.11 | 49.05 | 77.56 | 82.58 | 78.78 | 71.09 | 97.16 | 80.74 | 36.76 | 82.35 | 96.65 | 93.82 | 49.53 | 73.58 | 4.28 | 21.25 | 99.91 | 77.60 | 82.33 | 85.25 | 70.91 | 84.35 | 86.11 |
| *Methanobrevibacter* | Methanobacteriales | Methanobacteria | Euryarchaeota | 14.14 | 17.03 | 1.64 | 63.08 | 0.00 | 57.45 | 5.94 | 87.38 | 0.00 | 56.43 | 6.19 | 0.00 | 0.00 | 0.00 | 7.25 | 0.00 | 13.78 | 0.00 | 0.00 | 1.24 | 0.00 | 7.04 | 1.64 | 0.00 | 49.53 | 0.00 | 26.92 | 58.75 | 0.09 | 0.93 | 1.25 | 0.00 | 3.89 | 1.60 | 2.18 |
| VadinCA11 gut group | Thermoplasmatales | Thermoplasmata | Euryarchaeota | 12.16 | 13.41 | 6.72 | 24.82 | 12.14 | 0.27 | 17.48 | 12.62 | 18.91 | 3.22 | 15.74 | 48.99 | 26.94 | 49.52 | 5.51 | 5.19 | 4.23 | 5.86 | 0.00 | 5.43 | 63.24 | 2.43 | 1.25 | 0.00 | 0.00 | 4.51 | 1.68 | 18.75 | 0.00 | 7.70 | 6.38 | 6.09 | 6.50 | 5.06 | 8.61 |
| *Methanimicrococcus* | Methanosarcinales | Methanomicrobia | Euryarchaeota | 7.18 | 7.02 | 7.88 | 3.32 | 0.00 | 0.00 | 25.54 | 0.00 | 17.40 | 8.38 | 9.08 | 24.06 | 0.00 | 0.00 | 7.03 | 9.47 | 0.00 | 10.55 | 0.00 | 8.37 | 0.00 | 0.00 | 0.00 | 0.00 | 0.00 | 15.02 | 44.24 | 0.00 | 0.00 | 11.21 | 8.06 | 5.82 | 15.62 | 5.95 | 0.60 |
| *Methanoculleus* | Methanomicrobiales | Methanomicrobia | Euryarchaeota | 1.26 | 1.08 | 2.03 | 0.27 | 0.20 | 0.54 | 0.18 | 0.00 | 0.23 | 0.03 | 0.31 | 0.00 | 2.58 | 1.43 | 1.85 | 1.97 | 2.58 | 1.56 | 2.37 | 2.86 | 0.00 | 0.40 | 0.16 | 5.62 | 0.93 | 1.15 | 0.90 | 0.00 | 0.00 | 1.82 | 1.49 | 2.32 | 2.08 | 2.20 | 2.26 |
| *Methanobacterium* | Methanobacteriales | Methanobacteria | Euryarchaeota | 1.02 | 1.25 | 0.03 | 0.00 | 0.00 | 1.36 | 5.30 | 0.00 | 0.00 | 0.10 | 0.22 | 0.00 | 0.00 | 0.00 | 0.14 | 0.00 | 0.23 | 0.00 | 0.00 | 0.00 | 0.00 | 0.24 | 0.00 | 0.00 | 0.00 | 5.10 | 18.58 | 1.25 | 0.00 | 0.02 | 0.01 | 0.00 | 0.04 | 0.09 | 0.03 |
| *Methanospirillum* | Methanomicrobiales | Methanomicrobia | Euryarchaeota | 0.51 | 0.62 | 0.05 | 1.79 | 0.10 | 0.27 | 0.27 | 0.00 | 0.00 | 0.00 | 0.78 | 1.74 | 0.00 | 0.00 | 0.04 | 0.20 | 0.16 | 8.98 | 0.00 | 0.38 | 0.00 | 0.57 | 0.22 | 0.00 | 0.00 | 0.00 | 0.53 | 0.00 | 0.00 | 0.00 | 0.02 | 0.11 | 0.07 | 0.12 | 0.00 |
| *Methanosarcina* | Methanosarcinales | Methanomicrobia | Euryarchaeota | 0.25 | 0.22 | 0.38 | 0.00 | 0.00 | 0.00 | 0.03 | 0.00 | 0.00 | 0.06 | 0.07 | 0.58 | 0.00 | 0.00 | 0.46 | 0.48 | 0.00 | 0.00 | 0.00 | 0.44 | 0.00 | 0.00 | 0.00 | 0.56 | 0.00 | 0.54 | 2.41 | 0.00 | 0.00 | 0.57 | 0.27 | 0.29 | 0.77 | 0.36 | 0.03 |
| *Methanocalculus* | Methanomicrobiales | Methanomicrobia | Euryarchaeota | 0.14 | 0.17 | 0.03 | 0.27 | 0.00 | 0.54 | 0.06 | 0.00 | 0.00 | 0.00 | 0.22 | 0.58 | 0.00 | 0.00 | 0.03 | 0.03 | 0.16 | 1.95 | 0.00 | 0.19 | 0.00 | 0.08 | 0.08 | 0.00 | 0.00 | 0.00 | 0.17 | 0.00 | 0.00 | 0.02 | 0.02 | 0.04 | 0.02 | 0.07 | 0.00 |
| *Cenarchaeum* | Cenarchaeales | Incertae sedis | Thaumarchaeota | 0.12 | 0.15 | 0.00 | 0.00 | 0.00 | 0.00 | 0.00 | 0.00 | 0.00 | 0.00 | 0.00 | 0.00 | 0.00 | 0.00 | 0.00 | 0.00 | 0.00 | 0.00 | 0.00 | 0.00 | 0.00 | 3.89 | 0.00 | 0.00 | 0.00 | 0.00 | 0.00 | 0.00 | 0.00 | 0.00 | 0.00 | 0.00 | 0.00 | 0.00 | 0.00 |
| *Ca.* Nitrosoarchaeum | Nitrosopumilales | Incertae sedis | Thaumarchaeota | 0.09 | 0.11 | 0.00 | 0.00 | 0.00 | 0.00 | 0.00 | 0.00 | 0.00 | 0.00 | 0.00 | 0.00 | 0.00 | 0.00 | 0.00 | 0.00 | 0.00 | 0.00 | 0.00 | 0.00 | 0.00 | 2.91 | 0.00 | 0.00 | 0.00 | 0.00 | 0.00 | 0.00 | 0.00 | 0.00 | 0.00 | 0.00 | 0.00 | 0.00 | 0.00 |
| *Methanofollis* | Methanomicrobiales | Methanomicrobia | Euryarchaeota | 0.04 | 0.04 | 0.05 | 0.09 | 0.00 | 0.00 | 0.00 | 0.00 | 0.00 | 0.00 | 0.00 | 0.00 | 0.00 | 0.00 | 0.06 | 0.03 | 0.00 | 0.00 | 0.47 | 0.16 | 0.00 | 0.00 | 0.00 | 0.00 | 0.00 | 0.08 | 0.08 | 0.00 | 0.00 | 0.02 | 0.07 | 0.02 | 0.02 | 0.11 | 0.08 |
| *Methanosphaerula* | Methanomicrobiales | Methanomicrobia | Euryarchaeota | 0.03 | 0.02 | 0.05 | 0.00 | 0.00 | 0.00 | 0.00 | 0.00 | 0.00 | 0.00 | 0.00 | 0.00 | 0.37 | 0.00 | 0.02 | 0.05 | 0.00 | 0.00 | 0.00 | 0.08 | 0.00 | 0.00 | 0.01 | 0.00 | 0.00 | 0.03 | 0.06 | 0.00 | 0.00 | 0.03 | 0.06 | 0.04 | 0.00 | 0.08 | 0.06 |
| *Methanogenium* | Methanomicrobiales | Methanomicrobia | Euryarchaeota | 0.02 | 0.02 | 0.01 | 0.00 | 0.00 | 0.00 | 0.00 | 0.00 | 0.00 | 0.00 | 0.02 | 0.29 | 0.00 | 0.00 | 0.01 | 0.00 | 0.00 | 0.00 | 0.00 | 0.06 | 0.00 | 0.00 | 0.01 | 0.00 | 0.00 | 0.00 | 0.03 | 0.00 | 0.00 | 0.02 | 0.02 | 0.00 | 0.02 | 0.01 | 0.00 |
| *Methanothermobacter* | Methanobacteriales | Methanobacteria | Euryarchaeota | 0.01 | 0.01 | 0.01 | 0.00 | 0.00 | 0.00 | 0.00 | 0.00 | 0.00 | 0.00 | 0.00 | 0.00 | 0.00 | 0.00 | 0.02 | 0.00 | 0.00 | 0.00 | 0.00 | 0.00 | 0.00 | 0.00 | 0.00 | 0.00 | 0.00 | 0.00 | 0.14 | 0.00 | 0.00 | 0.02 | 0.01 | 0.00 | 0.04 | 0.01 | 0.00 |
| *Methanolinea* | Methanomicrobiales | Methanomicrobia | Euryarchaeota | 5E-3 | 0.01 | 0.00 | 0.09 | 0.00 | 0.00 | 0.00 | 0.00 | 0.00 | 0.00 | 0.00 | 0.00 | 0.00 | 0.00 | 0.02 | 0.02 | 0.00 | 0.00 | 0.00 | 0.01 | 0.00 | 0.00 | 0.00 | 0.00 | 0.00 | 0.00 | 0.00 | 0.00 | 0.00 | 0.00 | 0.01 | 0.00 | 0.00 | 0.01 | 0.00 |
| *Ca.* Nitrosocaldus | Incertae sedis | Incertae sedis | Thaumarchaeota | 3E-3 | 0.00 | 2E-2 | 0.00 | 0.00 | 0.00 | 0.00 | 0.00 | 0.00 | 0.00 | 0.00 | 0.00 | 0.00 | 0.00 | 0.00 | 0.00 | 0.00 | 0.00 | 0.00 | 0.00 | 0.00 | 0.00 | 0.00 | 0.00 | 0.00 | 0.00 | 0.00 | 0.00 | 0.00 | 0.06 | 0.00 | 0.00 | 0.00 | 0.00 | 0.03 |
| *Methanoregula* | Methanomicrobiales | Methanomicrobia | Euryarchaeota | 3E-3 | 3E-3 | 0.00 | 0.00 | 0.00 | 0.00 | 0.00 | 0.00 | 0.00 | 0.00 | 0.00 | 0.00 | 0.00 | 0.00 | 0.00 | 0.00 | 0.08 | 0.00 | 0.00 | 0.01 | 0.00 | 0.00 | 0.00 | 0.00 | 0.00 | 0.00 | 0.00 | 0.00 | 0.00 | 0.00 | 0.00 | 0.00 | 0.00 | 0.00 | 0.00 |
| Marine archaeal group I | Incertae sedis | Incertae sedis | Thaumarchaeota | 3E-3 | 3E-3 | 0.00 | 0.00 | 0.00 | 0.00 | 0.00 | 0.00 | 0.00 | 0.00 | 0.00 | 0.00 | 0.00 | 0.00 | 0.00 | 0.00 | 0.00 | 0.00 | 0.00 | 0.00 | 0.00 | 0.08 | 0.00 | 0.00 | 0.00 | 0.00 | 0.00 | 0.00 | 0.00 | 0.00 | 0.00 | 0.00 | 0.00 | 0.00 | 0.00 |
| *Methanoplanus* | Methanomicrobiales | Methanomicrobia | Euryarchaeota | 7E-4 | 9E-4 | 0.00 | 0.00 | 0.00 | 0.00 | 0.00 | 0.00 | 0.00 | 0.00 | 0.02 | 0.00 | 0.00 | 0.00 | 0.00 | 0.00 | 0.00 | 0.00 | 0.00 | 0.00 | 0.00 | 0.00 | 0.00 | 0.00 | 0.00 | 0.00 | 0.00 | 0.00 | 0.00 | 0.00 | 0.00 | 0.00 | 0.00 | 0.00 | 0.00 |
| *Methanomicrobium* | Methanomicrobiales | Methanomicrobia | Euryarchaeota | 5E-4 | 7E-4 | 0.00 | 0.00 | 0.00 | 0.00 | 0.00 | 0.00 | 0.00 | 0.00 | 0.00 | 0.00 | 0.00 | 0.00 | 0.01 | 0.00 | 0.00 | 0.00 | 0.00 | 0.01 | 0.00 | 0.00 | 0.00 | 0.00 | 0.00 | 0.00 | 0.00 | 0.00 | 0.00 | 0.00 | 0.00 | 0.00 | 0.00 | 0.00 | 0.00 |
| *Methanolobus* | Methanosarcinales | Methanomicrobia | Euryarchaeota | 5E-4 | 7E-4 | 0.00 | 0.00 | 0.00 | 0.00 | 0.00 | 0.00 | 0.00 | 0.00 | 0.00 | 0.00 | 0.00 | 0.00 | 0.00 | 0.00 | 0.00 | 0.00 | 0.00 | 0.02 | 0.00 | 0.00 | 0.00 | 0.00 | 0.00 | 0.00 | 0.00 | 0.00 | 0.00 | 0.00 | 0.00 | 0.00 | 0.00 | 0.00 | 0.00 |
| *Methermicoccus* | Methanosarcinales | Methanomicrobia | Euryarchaeota | 3E-4 | 0.00 | 2E-3 | 0.00 | 0.00 | 0.00 | 0.00 | 0.00 | 0.00 | 0.00 | 0.00 | 0.00 | 0.00 | 0.00 | 0.00 | 0.00 | 0.00 | 0.00 | 0.00 | 0.00 | 0.00 | 0.00 | 0.00 | 0.00 | 0.00 | 0.00 | 0.00 | 0.00 | 0.00 | 0.00 | 0.00 | 0.01 | 0.00 | 0.00 | 0.00 |
| *Methanosalsum* | Methanosarcinales | Methanomicrobia | Euryarchaeota | 3E-4 | 3E-4 | 0.00 | 0.00 | 0.00 | 0.00 | 0.00 | 0.00 | 0.00 | 0.00 | 0.00 | 0.00 | 0.00 | 0.00 | 0.00 | 0.00 | 0.00 | 0.00 | 0.00 | 0.01 | 0.00 | 0.00 | 0.00 | 0.00 | 0.00 | 0.00 | 0.00 | 0.00 | 0.00 | 0.00 | 0.00 | 0.00 | 0.00 | 0.00 | 0.00 |
| *Methanosphaera* | Methanobacteriales | Methanobacteria | Euryarchaeota | 3E-4 | 0.00 | 1E-3 | 0.00 | 0.00 | 0.00 | 0.00 | 0.00 | 0.00 | 0.00 | 0.00 | 0.00 | 0.00 | 0.00 | 0.00 | 0.00 | 0.00 | 0.00 | 0.00 | 0.00 | 0.00 | 0.00 | 0.00 | 0.00 | 0.00 | 0.00 | 0.00 | 0.00 | 0.00 | 0.00 | 0.00 | 0.00 | 0.00 | 0.00 | 0.01 |

**Notes:**

Genera are arranged in decreasing order. For sample codes, see Table 1.

M_tot_ = mean relative abundance averaged over all samples; M_Cil_ = mean relative abundance averaged over hindgut ciliate samples; M_Int_ = mean relative abundance averaged over all ciliate-free hindgut samples.

**Supplementary Table S3.** Relative abundances (in %) of bacterial phyla in the samples studied.

| **Phylum** | **M_tot_** | **M_Cil_** | **M_Int_** | **07-Pac-V** | **08-Paa-T** | **09-Paa-T** | **10-Paa-T** | **02-Pac-K** | **03-Pac-K** | **14-Paa-T** | **15-Paa-T** | **16-Pac-V** | **17-Pac-V** | **21-Pac-V** | **25-Paa-T** | **26-Paa-T** | **29-Paa-T** | **31-Paa-T** | **06-Pac-V** | **23-Pac-V** | **24-Pac-V** | **04-Pac-K** | **11-Paa-T** | **18-Pac-V** | **22-Pac-V** | **27-Paa-T** | **28-Paa-T** | **30-Paa-T** | **13-Paa-T** | **19-Pac-K** | **I10-Pac-V** | **I11-Pac-V** | **I13-Pac-V** | **I15-Pac-V** | **I20-Paa-T** | **I30-Pac-K** |
| --- | --- | --- | --- | --- | --- | --- | --- | --- | --- | --- | --- | --- | --- | --- | --- | --- | --- | --- | --- | --- | --- | --- | --- | --- | --- | --- | --- | --- | --- | --- | --- | --- | --- | --- | --- | --- |
| Proteobacteria | 60.20 | 70.05 | 15.87 | 57.40 | 71.21 | 73.08 | 54.97 | 69.51 | 80.09 | 64.62 | 68.42 | 56.46 | 63.61 | 89.47 | 80.51 | 68.04 | 74.08 | 57.98 | 73.19 | 93.71 | 54.31 | 75.54 | 71.02 | 59.69 | 93.75 | 78.88 | 58.04 | 93.03 | 48.08 | 62.73 | 14.09 | 20.23 | 21.67 | 19.92 | 6.62 | 12.68 |
| Bacteroidetes | 10.63 | 5.78 | 32.44 | 11.16 | 2.32 | 2.62 | 1.73 | 11.75 | 3.15 | 14.06 | 3.38 | 14.88 | 4.08 | 3.17 | 5.68 | 7.05 | 10.37 | 1.84 | 2.46 | 1.69 | 14.56 | 3.01 | 0.27 | 8.90 | 1.91 | 10.76 | 6.33 | 1.81 | 1.67 | 5.48 | 24.21 | 39.98 | 38.54 | 29.90 | 23.62 | 38.41 |
| Firmicutes | 8.24 | 8.72 | 6.11 | 11.28 | 8.20 | 14.03 | 10.34 | 7.72 | 8.81 | 6.58 | 4.66 | 6.30 | 3.72 | 2.63 | 3.71 | 12.65 | 3.61 | 28.30 | 12.74 | 3.02 | 4.23 | 9.40 | 3.38 | 7.69 | 2.77 | 1.52 | 23.45 | 1.68 | 22.81 | 10.13 | 3.90 | 3.55 | 8.48 | 11.70 | 4.03 | 5.00 |
| Lentisphaerae | 6.46 | 3.85 | 18.21 | 3.31 | 3.94 | 0.97 | 22.13 | 0.97 | 0.00 | 2.51 | 13.54 | 3.17 | 1.85 | 0.00 | 1.03 | 3.27 | 0.66 | 1.07 | 0.00 | 0.05 | 4.55 | 0.77 | 13.33 | 3.06 | 0.00 | 0.49 | 1.70 | 0.37 | 20.76 | 0.52 | 23.98 | 13.63 | 12.43 | 13.89 | 27.00 | 18.36 |
| Actinobacteria | 5.09 | 6.01 | 0.95 | 5.32 | 8.56 | 7.25 | 1.65 | 1.66 | 6.80 | 0.66 | 1.85 | 2.25 | 25.66 | 4.17 | 6.22 | 2.89 | 3.68 | 8.66 | 7.36 | 1.54 | 2.62 | 9.83 | 3.94 | 13.83 | 1.57 | 4.11 | 3.48 | 2.22 | 4.83 | 19.65 | 0.24 | 1.58 | 0.96 | 2.37 | 0.12 | 0.40 |
| Verrucomicrobia | 2.32 | 1.12 | 7.77 | 5.27 | 2.22 | 0.53 | 2.06 | 2.50 | 0.19 | 1.07 | 1.92 | 4.42 | 0.00 | 0.02 | 0.00 | 1.81 | 1.55 | 0.01 | 0.00 | 0.00 | 2.46 | 0.00 | 0.76 | 1.08 | 0.00 | 0.08 | 0.94 | 0.51 | 0.69 | 0.03 | 10.92 | 5.94 | 5.73 | 5.98 | 6.23 | 11.79 |
| Elusimicrobia | 2.07 | 1.34 | 5.31 | 0.15 | 0.66 | 0.00 | 5.55 | 1.31 | 0.00 | 2.12 | 3.83 | 1.08 | 0.00 | 0.00 | 2.33 | 0.79 | 0.00 | 0.40 | 0.08 | 0.00 | 11.21 | 1.11 | 4.25 | 1.12 | 0.00 | 0.00 | 0.00 | 0.00 | 0.30 | 0.00 | 2.46 | 1.47 | 0.78 | 2.79 | 15.24 | 9.11 |
| Tenericutes | 1.87 | 0.69 | 7.14 | 0.00 | 0.00 | 0.00 | 0.00 | 1.48 | 0.00 | 1.31 | 0.00 | 4.28 | 0.00 | 0.53 | 0.00 | 0.67 | 3.87 | 0.25 | 1.88 | 0.00 | 1.93 | 0.00 | 0.00 | 0.57 | 0.00 | 0.12 | 0.40 | 0.12 | 0.65 | 0.69 | 11.15 | 8.09 | 3.92 | 6.63 | 10.48 | 2.55 |
| Spirochaetes | 1.06 | 0.48 | 3.65 | 2.40 | 0.00 | 0.00 | 0.00 | 0.74 | 0.00 | 0.84 | 0.00 | 3.51 | 0.00 | 0.00 | 0.00 | 0.55 | 0.91 | 0.01 | 0.00 | 0.00 | 1.26 | 0.00 | 0.00 | 2.70 | 0.00 | 0.00 | 0.13 | 0.00 | 0.00 | 0.04 | 4.94 | 4.12 | 5.34 | 5.46 | 1.84 | 0.22 |
| Planctomycetes | 0.73 | 0.55 | 1.52 | 0.01 | 0.07 | 0.49 | 0.33 | 0.01 | 0.00 | 0.19 | 0.52 | 0.16 | 0.19 | 0.00 | 0.14 | 0.82 | 0.03 | 1.39 | 0.54 | 0.00 | 1.14 | 0.01 | 1.01 | 0.35 | 0.00 | 3.77 | 3.54 | 0.04 | 0.19 | 0.00 | 1.90 | 0.64 | 1.25 | 0.95 | 3.79 | 0.63 |
| Synergistetes | 0.67 | 0.79 | 0.17 | 1.97 | 1.27 | 0.00 | 0.79 | 0.64 | 0.96 | 4.22 | 1.14 | 3.11 | 0.00 | 0.00 | 0.26 | 1.46 | 0.82 | 0.09 | 0.00 | 0.00 | 1.42 | 0.34 | 0.00 | 1.01 | 0.00 | 0.27 | 0.63 | 0.22 | 0.00 | 0.57 | 0.28 | 0.21 | 0.04 | 0.37 | 0.14 | 0.00 |
| *Ca.* Saccharibacteria | 0.17 | 0.12 | 0.43 | 0.00 | 0.85 | 0.57 | 0.00 | 0.00 | 0.00 | 0.89 | 0.09 | 0.20 | 0.00 | 0.00 | 0.00 | 0.01 | 0.12 | 0.00 | 0.40 | 0.00 | 0.00 | 0.00 | 0.00 | 0.00 | 0.00 | 0.00 | 0.00 | 0.00 | 0.00 | 0.00 | 0.51 | 0.43 | 0.14 | 0.03 | 0.82 | 0.67 |
| Deinococcus-Thermus | 0.08 | 0.07 | 0.13 | 0.71 | 0.00 | 0.00 | 0.00 | 0.14 | 0.00 | 0.00 | 0.00 | 0.00 | 0.00 | 0.00 | 0.00 | 0.00 | 0.00 | 0.00 | 0.00 | 0.00 | 0.00 | 0.00 | 0.00 | 0.00 | 0.00 | 0.00 | 1.07 | 0.00 | 0.00 | 0.00 | 0.04 | 0.00 | 0.72 | 0.00 | 0.00 | 0.00 |
| Fusobacteria | 0.07 | 0.06 | 0.15 | 1.02 | 0.00 | 0.00 | 0.00 | 0.00 | 0.00 | 0.00 | 0.00 | 0.19 | 0.00 | 0.00 | 0.00 | 0.00 | 0.30 | 0.00 | 0.00 | 0.00 | 0.00 | 0.00 | 0.00 | 0.00 | 0.00 | 0.00 | 0.00 | 0.00 | 0.00 | 0.00 | 0.88 | 0.00 | 0.00 | 0.00 | 0.00 | 0.00 |
| BRC1 | 0.07 | 0.07 | 0.04 | 0.00 | 0.00 | 0.00 | 0.28 | 0.00 | 0.00 | 0.00 | 0.00 | 0.00 | 0.00 | 0.00 | 0.13 | 0.00 | 0.00 | 0.00 | 1.22 | 0.00 | 0.32 | 0.00 | 0.00 | 0.00 | 0.00 | 0.00 | 0.00 | 0.00 | 0.00 | 0.00 | 0.11 | 0.00 | 0.00 | 0.00 | 0.05 | 0.09 |
| Gemmatimonadetes | 0.06 | 0.07 | 0.00 | 0.00 | 0.42 | 0.46 | 0.17 | 0.00 | 0.00 | 0.00 | 0.65 | 0.00 | 0.00 | 0.00 | 0.00 | 0.00 | 0.00 | 0.00 | 0.00 | 0.00 | 0.00 | 0.00 | 0.24 | 0.00 | 0.00 | 0.00 | 0.00 | 0.00 | 0.00 | 0.00 | 0.00 | 0.00 | 0.00 | 0.00 | 0.00 | 0.00 |
| Armatimonadetes | 0.05 | 0.06 | 0.00 | 0.00 | 0.29 | 0.00 | 0.00 | 0.00 | 0.00 | 0.00 | 0.00 | 0.00 | 0.00 | 0.00 | 0.00 | 0.00 | 0.00 | 0.00 | 0.00 | 0.00 | 0.00 | 0.00 | 1.15 | 0.00 | 0.00 | 0.00 | 0.28 | 0.00 | 0.00 | 0.00 | 0.00 | 0.00 | 0.00 | 0.00 | 0.00 | 0.00 |
| *Candidate* division WPS-1 | 0.05 | 0.06 | 0.00 | 0.00 | 0.00 | 0.00 | 0.00 | 0.00 | 0.00 | 0.00 | 0.00 | 0.00 | 0.88 | 0.00 | 0.00 | 0.00 | 0.00 | 0.00 | 0.00 | 0.00 | 0.00 | 0.00 | 0.64 | 0.00 | 0.00 | 0.00 | 0.00 | 0.00 | 0.00 | 0.17 | 0.00 | 0.00 | 0.00 | 0.00 | 0.00 | 0.00 |
| Microgenomates | 0.05 | 0.06 | 0.00 | 0.00 | 0.00 | 0.00 | 0.00 | 1.53 | 0.00 | 0.00 | 0.00 | 0.00 | 0.00 | 0.00 | 0.00 | 0.00 | 0.00 | 0.00 | 0.00 | 0.00 | 0.00 | 0.00 | 0.00 | 0.00 | 0.00 | 0.00 | 0.00 | 0.00 | 0.00 | 0.00 | 0.00 | 0.00 | 0.00 | 0.00 | 0.00 | 0.00 |
| Nitrospirae | 0.02 | 0.03 | 0.00 | 0.00 | 0.00 | 0.00 | 0.00 | 0.00 | 0.00 | 0.78 | 0.00 | 0.00 | 0.00 | 0.00 | 0.00 | 0.00 | 0.00 | 0.00 | 0.00 | 0.00 | 0.00 | 0.00 | 0.00 | 0.00 | 0.00 | 0.00 | 0.00 | 0.00 | 0.00 | 0.00 | 0.00 | 0.00 | 0.00 | 0.00 | 0.00 | 0.00 |
| SR1 | 0.02 | 0.01 | 0.10 | 0.00 | 0.00 | 0.00 | 0.00 | 0.03 | 0.00 | 0.13 | 0.00 | 0.00 | 0.00 | 0.00 | 0.00 | 0.00 | 0.00 | 0.00 | 0.00 | 0.00 | 0.00 | 0.00 | 0.00 | 0.00 | 0.00 | 0.00 | 0.00 | 0.00 | 0.00 | 0.00 | 0.34 | 0.13 | 0.00 | 0.00 | 0.02 | 0.09 |
| Acidobacteria | 0.01 | 0.00 | 0.01 | 0.00 | 0.00 | 0.00 | 0.00 | 0.00 | 0.00 | 0.00 | 0.00 | 0.00 | 0.00 | 0.00 | 0.00 | 0.00 | 0.00 | 0.00 | 0.13 | 0.00 | 0.00 | 0.00 | 0.00 | 0.00 | 0.00 | 0.00 | 0.00 | 0.00 | 0.00 | 0.00 | 0.06 | 0.00 | 0.00 | 0.00 | 0.00 | 0.00 |

**Notes:**

Phyla are arranged in decreasing order. For sample codes, see Table 1.

M_tot_ = mean relative abundance averaged over all samples; M_Cil_ = mean relative abundance averaged over hindgut ciliate samples; M_Int_ = mean relative abundance averaged over all ciliate-free hindgut samples.

**Supplementary Table S4.** Relative abundances (in %) of 30 most common bacterial genera in the samples studied.

| **Genus** | **M_tot_** | **M_Cil_** | **M_Int_** | **07-Pac-V** | **08-Paa-T** | **09-Paa-T** | **10-Paa-T** | **02-Pac-K** | **03-Pac-K** | **14-Paa-T** | **15-Paa-T** | **16-Pac-V** | **17-Pac-V** | **21-Pac-V** | **25-Paa-T** | **26-Paa-T** | **29-Paa-T** | **31-Paa-T** | **06-Pac-V** | **23-Pac-V** | **24-Pac-V** | **04-Pac-K** | **11-Paa-T** | **18-Pac-V** | **22-Pac-V** | **27-Paa-T** | **28-Paa-T** | **30-Paa-T** | **13-Paa-T** | **19-Pac-K** | **I10-Pac-V** | **I11-Pac-V** | **I13-Pac-V** | **I15-Pac-V** | **I20-Paa-T** | **I30-Pac-K** |
| --- | --- | --- | --- | --- | --- | --- | --- | --- | --- | --- | --- | --- | --- | --- | --- | --- | --- | --- | --- | --- | --- | --- | --- | --- | --- | --- | --- | --- | --- | --- | --- | --- | --- | --- | --- | --- |
| *Pseudomonas* | 33.54 | 40.98 | 0.07 | 35.64 | 27.18 | 40.61 | 16.73 | 31.30 | 52.77 | 38.66 | 40.32 | 23.51 | 33.15 | 67.40 | 50.48 | 43.04 | 55.60 | 38.50 | 48.42 | 67.78 | 31.29 | 51.21 | 30.71 | 35.54 | 56.33 | 47.37 | 28.84 | 69.52 | 15.37 | 29.16 | 0.04 | 0.06 | 0.02 | 0.00 | 0.19 | 0.09 |
| *Victivallis* | 6.46 | 3.85 | 18.21 | 3.31 | 3.94 | 0.97 | 22.13 | 0.97 | 0.00 | 2.51 | 13.54 | 3.17 | 1.85 | 0.00 | 1.03 | 3.27 | 0.66 | 1.07 | 0.00 | 0.05 | 4.55 | 0.77 | 13.33 | 3.06 | 0.00 | 0.49 | 1.70 | 0.37 | 20.76 | 0.52 | 23.98 | 13.63 | 12.43 | 13.89 | 27.00 | 18.36 |
| *Staphylococcus* | 4.08 | 4.63 | 1.58 | 5.62 | 2.51 | 4.68 | 8.25 | 0.28 | 5.21 | 2.04 | 3.01 | 3.77 | 1.46 | 0.02 | 1.27 | 0.07 | 0.09 | 24.80 | 1.20 | 2.86 | 1.39 | 1.48 | 1.86 | 3.79 | 1.59 | 0.91 | 23.20 | 1.09 | 12.86 | 9.81 | 0.09 | 0.51 | 2.55 | 5.51 | 0.12 | 0.67 |
| *Alistipes* | 3.39 | 1.47 | 12.02 | 2.25 | 0.93 | 0.00 | 0.54 | 4.78 | 1.17 | 8.06 | 1.59 | 3.20 | 1.19 | 0.01 | 0.99 | 2.82 | 2.61 | 0.00 | 0.00 | 0.00 | 5.53 | 0.00 | 0.00 | 0.99 | 0.10 | 0.59 | 0.64 | 0.36 | 0.21 | 1.01 | 12.42 | 10.27 | 12.64 | 8.80 | 11.88 | 16.12 |
| *Achromobacter* | 3.25 | 3.51 | 2.08 | 2.06 | 0.45 | 0.77 | 0.04 | 0.95 | 9.64 | 2.55 | 1.58 | 2.90 | 3.66 | 4.00 | 5.44 | 1.78 | 8.16 | 4.62 | 2.40 | 8.11 | 4.07 | 5.40 | 1.46 | 0.21 | 9.05 | 1.93 | 1.69 | 5.91 | 1.89 | 4.12 | 0.75 | 3.29 | 3.53 | 2.39 | 0.31 | 2.23 |
| *Hydrotalea* | 2.03 | 0.98 | 6.75 | 3.72 | 0.00 | 0.00 | 0.57 | 0.61 | 0.00 | 0.71 | 0.00 | 0.01 | 1.58 | 3.01 | 3.17 | 1.61 | 0.00 | 0.19 | 0.69 | 0.00 | 2.05 | 2.69 | 0.00 | 1.13 | 1.10 | 0.64 | 0.83 | 1.10 | 0.00 | 1.13 | 2.81 | 6.66 | 17.02 | 5.63 | 0.99 | 7.37 |
| *Delftia* | 2.00 | 2.31 | 0.58 | 1.53 | 4.75 | 2.89 | 1.07 | 5.18 | 1.24 | 3.73 | 6.44 | 1.19 | 1.90 | 3.19 | 4.29 | 2.05 | 0.00 | 0.92 | 0.00 | 2.10 | 0.44 | 2.04 | 2.36 | 4.36 | 1.87 | 0.24 | 1.86 | 2.31 | 1.57 | 2.91 | 0.11 | 0.81 | 1.03 | 1.07 | 0.07 | 0.40 |
| *Stenotrophomonas* | 1.99 | 2.30 | 0.59 | 5.49 | 0.00 | 6.99 | 1.54 | 3.21 | 8.37 | 1.17 | 3.52 | 1.85 | 2.59 | 0.00 | 1.31 | 1.25 | 2.32 | 1.09 | 0.90 | 1.60 | 1.39 | 4.99 | 4.27 | 0.79 | 2.12 | 1.90 | 1.67 | 0.00 | 0.79 | 1.05 | 0.08 | 0.13 | 2.62 | 0.32 | 0.07 | 0.31 |
| *Ralstonia* | 1.76 | 2.15 | 0.03 | 0.79 | 6.92 | 1.11 | 6.28 | 0.15 | 0.00 | 2.92 | 2.06 | 0.00 | 0.00 | 1.56 | 8.34 | 4.82 | 0.00 | 0.52 | 0.00 | 3.02 | 1.83 | 1.69 | 3.28 | 0.00 | 2.38 | 0.79 | 1.80 | 0.00 | 7.70 | 0.00 | 0.00 | 0.02 | 0.02 | 0.00 | 0.02 | 0.09 |
| *Acholeplasma* | 1.71 | 0.69 | 6.29 | 0.00 | 0.00 | 0.00 | 0.00 | 1.48 | 0.00 | 1.31 | 0.00 | 4.28 | 0.00 | 0.53 | 0.00 | 0.67 | 3.87 | 0.25 | 1.88 | 0.00 | 1.93 | 0.00 | 0.00 | 0.57 | 0.00 | 0.12 | 0.40 | 0.12 | 0.65 | 0.69 | 9.46 | 7.01 | 3.02 | 5.43 | 10.38 | 2.46 |
| *Nocardioides* | 1.66 | 2.01 | 0.11 | 0.82 | 3.41 | 3.64 | 0.24 | 0.15 | 4.19 | 0.15 | 0.36 | 0.76 | 8.16 | 1.45 | 1.23 | 0.50 | 0.99 | 0.16 | 3.21 | 0.78 | 0.57 | 3.73 | 0.36 | 6.65 | 0.15 | 0.15 | 0.43 | 0.14 | 0.30 | 11.49 | 0.04 | 0.17 | 0.38 | 0.03 | 0.02 | 0.00 |
| *Desulfovibrio* | 1.53 | 0.72 | 5.22 | 2.86 | 0.56 | 1.22 | 3.39 | 0.28 | 0.00 | 0.71 | 3.26 | 0.97 | 0.73 | 0.08 | 0.83 | 1.50 | 0.71 | 0.20 | 0.10 | 0.00 | 0.41 | 0.13 | 0.70 | 0.04 | 0.00 | 0.32 | 0.17 | 0.12 | 0.02 | 0.00 | 8.61 | 7.11 | 4.16 | 8.32 | 2.46 | 0.63 |
| *Enhydrobacter* | 1.51 | 1.85 | 0.00 | 0.69 | 4.50 | 0.61 | 10.26 | 0.58 | 2.10 | 0.00 | 1.53 | 0.01 | 9.31 | 0.65 | 0.00 | 0.00 | 0.00 | 0.49 | 2.06 | 0.00 | 0.00 | 0.00 | 15.74 | 1.15 | 0.30 | 0.00 | 0.00 | 0.00 | 0.00 | 0.00 | 0.00 | 0.00 | 0.00 | 0.00 | 0.00 | 0.00 |
| *Elusimicrobium* | 1.48 | 1.08 | 3.27 | 0.15 | 0.66 | 0.00 | 5.55 | 0.61 | 0.00 | 2.12 | 3.83 | 0.00 | 0.00 | 0.00 | 0.93 | 0.37 | 0.00 | 0.00 | 0.08 | 0.00 | 9.28 | 1.11 | 4.25 | 0.00 | 0.00 | 0.00 | 0.00 | 0.00 | 0.30 | 0.00 | 1.95 | 0.85 | 0.78 | 1.30 | 11.06 | 3.71 |
| *Acinetobacter* | 1.48 | 1.77 | 0.15 | 0.01 | 6.93 | 0.00 | 0.86 | 0.31 | 0.01 | 1.75 | 0.68 | 12.76 | 3.67 | 0.36 | 0.00 | 0.00 | 0.00 | 0.74 | 3.10 | 0.00 | 0.00 | 0.00 | 0.73 | 0.25 | 9.11 | 0.74 | 2.44 | 0.00 | 1.15 | 2.29 | 0.00 | 0.90 | 0.00 | 0.00 | 0.02 | 0.00 |
| *Janibacter* | 1.45 | 1.77 | 0.02 | 2.94 | 3.47 | 2.72 | 0.00 | 0.22 | 1.08 | 0.22 | 0.00 | 0.64 | 12.26 | 0.82 | 0.11 | 0.00 | 0.70 | 0.00 | 2.05 | 0.16 | 0.00 | 4.89 | 0.00 | 6.81 | 0.07 | 0.02 | 0.10 | 0.35 | 0.78 | 7.41 | 0.00 | 0.00 | 0.00 | 0.10 | 0.02 | 0.00 |
| *Tepidiphilus* | 1.45 | 1.77 | 0.00 | 1.46 | 1.43 | 6.68 | 0.00 | 0.00 | 0.00 | 0.70 | 0.00 | 0.52 | 0.96 | 4.70 | 0.00 | 1.48 | 1.60 | 0.00 | 2.97 | 0.00 | 3.47 | 0.00 | 1.87 | 5.84 | 0.92 | 9.10 | 0.00 | 0.96 | 0.68 | 2.41 | 0.00 | 0.00 | 0.00 | 0.00 | 0.00 | 0.00 |
| *Bacteroides* | 1.24 | 0.84 | 3.06 | 1.57 | 0.01 | 0.00 | 0.05 | 1.09 | 0.00 | 0.26 | 0.21 | 7.15 | 0.03 | 0.03 | 0.50 | 1.00 | 1.10 | 0.11 | 1.25 | 0.00 | 2.53 | 0.00 | 0.00 | 4.45 | 0.00 | 0.44 | 0.30 | 0.10 | 0.00 | 0.43 | 2.87 | 2.82 | 1.99 | 3.97 | 2.70 | 4.02 |
| *Treponema* | 1.02 | 0.47 | 3.50 | 2.40 | 0.00 | 0.00 | 0.00 | 0.62 | 0.00 | 0.73 | 0.00 | 3.49 | 0.00 | 0.00 | 0.00 | 0.55 | 0.91 | 0.01 | 0.00 | 0.00 | 1.23 | 0.00 | 0.00 | 2.70 | 0.00 | 0.00 | 0.12 | 0.00 | 0.00 | 0.00 | 4.69 | 3.89 | 5.12 | 5.43 | 1.69 | 0.18 |
| Subdivision 5 genera | 1.01 | 0.40 | 3.78 | 1.00 | 0.55 | 0.00 | 0.49 | 1.65 | 0.00 | 0.67 | 0.00 | 1.63 | 0.00 | 0.00 | 0.00 | 0.76 | 1.55 | 0.00 | 0.00 | 0.00 | 1.20 | 0.00 | 0.00 | 0.00 | 0.00 | 0.00 | 0.74 | 0.51 | 0.00 | 0.00 | 3.40 | 2.35 | 1.25 | 1.78 | 4.18 | 9.74 |
| *Burkholderia* | 0.94 | 1.02 | 0.55 | 0.01 | 0.02 | 0.01 | 0.02 | 0.02 | 0.02 | 0.01 | 0.00 | 0.01 | 0.01 | 0.62 | 1.08 | 4.58 | 0.42 | 4.42 | 0.01 | 1.71 | 0.54 | 0.02 | 0.01 | 0.01 | 3.52 | 3.92 | 3.58 | 3.04 | 0.01 | 0.01 | 0.56 | 0.26 | 0.71 | 0.53 | 0.60 | 0.67 |
| *Streptococcus* | 0.86 | 1.01 | 0.20 | 1.77 | 1.03 | 3.20 | 0.00 | 0.83 | 0.00 | 0.00 | 0.00 | 0.00 | 0.00 | 0.00 | 0.08 | 9.55 | 0.12 | 1.20 | 4.64 | 0.00 | 0.03 | 2.75 | 0.00 | 0.00 | 0.00 | 0.01 | 0.00 | 0.02 | 1.94 | 0.00 | 0.04 | 0.04 | 0.11 | 0.98 | 0.02 | 0.00 |
| *Bradyrhizobium* | 0.83 | 0.62 | 1.78 | 0.55 | 0.00 | 4.06 | 0.00 | 0.00 | 0.66 | 0.90 | 0.00 | 0.00 | 0.00 | 0.02 | 0.00 | 1.45 | 0.94 | 0.36 | 1.91 | 0.08 | 1.33 | 0.02 | 0.00 | 0.68 | 0.00 | 0.21 | 2.06 | 0.87 | 0.63 | 0.00 | 0.64 | 2.61 | 3.36 | 2.29 | 0.31 | 1.47 |
| *Opitutus* | 0.81 | 0.36 | 2.81 | 4.27 | 0.21 | 0.00 | 0.00 | 0.60 | 0.00 | 0.00 | 0.00 | 2.20 | 0.00 | 0.02 | 0.00 | 0.84 | 0.00 | 0.00 | 0.00 | 0.00 | 1.07 | 0.00 | 0.00 | 0.02 | 0.00 | 0.00 | 0.01 | 0.00 | 0.45 | 0.02 | 5.55 | 2.65 | 3.36 | 3.14 | 1.13 | 1.03 |
| *Propionibacterium* | 0.71 | 0.78 | 0.42 | 0.42 | 0.14 | 0.26 | 0.97 | 0.11 | 0.12 | 0.03 | 0.27 | 0.50 | 0.24 | 1.74 | 3.01 | 0.42 | 1.67 | 2.56 | 0.83 | 0.05 | 1.26 | 0.42 | 0.72 | 0.30 | 0.56 | 1.39 | 1.05 | 1.30 | 0.31 | 0.28 | 0.02 | 0.00 | 0.58 | 1.49 | 0.02 | 0.40 |
| *Dysgonomonas* | 0.66 | 0.47 | 1.53 | 2.40 | 0.85 | 0.00 | 0.00 | 0.82 | 0.23 | 2.27 | 0.00 | 0.40 | 0.38 | 0.01 | 0.76 | 0.04 | 0.94 | 0.07 | 0.00 | 0.00 | 2.49 | 0.00 | 0.00 | 0.02 | 0.00 | 0.33 | 0.10 | 0.17 | 0.31 | 0.00 | 1.67 | 1.07 | 1.10 | 1.38 | 1.79 | 2.19 |
| *Cloacibacillus* | 0.63 | 0.73 | 0.17 | 1.12 | 1.27 | 0.00 | 0.79 | 0.59 | 0.78 | 4.07 | 1.14 | 3.11 | 0.00 | 0.00 | 0.26 | 1.45 | 0.82 | 0.09 | 0.00 | 0.00 | 1.42 | 0.34 | 0.00 | 0.77 | 0.00 | 0.27 | 0.63 | 0.22 | 0.00 | 0.57 | 0.24 | 0.21 | 0.04 | 0.37 | 0.14 | 0.00 |
| *Ca.* Endomicrobium | 0.58 | 0.26 | 2.03 | 0.00 | 0.00 | 0.00 | 0.00 | 0.71 | 0.00 | 0.00 | 0.00 | 1.08 | 0.00 | 0.00 | 1.40 | 0.42 | 0.00 | 0.40 | 0.00 | 0.00 | 1.93 | 0.00 | 0.00 | 1.12 | 0.00 | 0.00 | 0.00 | 0.00 | 0.00 | 0.00 | 0.51 | 0.62 | 0.00 | 1.49 | 4.18 | 5.40 |
| *Aeromonas* | 0.54 | 40.98 | 0.07 | 0.00 | 3.02 | 0.97 | 4.39 | 1.26 | 0.00 | 2.47 | 2.27 | 0.00 | 0.00 | 0.02 | 0.04 | 0.01 | 0.02 | 0.03 | 1.91 | 0.00 | 0.00 | 0.00 | 0.00 | 0.00 | 0.00 | 0.02 | 0.00 | 0.02 | 1.13 | 0.00 | 0.00 | 0.00 | 0.00 | 0.00 | 0.00 | 0.09 |
| *Pelomonas* | 0.53 | 3.85 | 18.21 | 0.00 | 0.59 | 0.69 | 0.00 | 0.82 | 1.63 | 0.86 | 0.76 | 0.75 | 0.00 | 0.48 | 0.17 | 0.12 | 0.07 | 0.31 | 0.77 | 0.00 | 0.00 | 2.84 | 1.40 | 1.09 | 0.42 | 0.12 | 0.20 | 0.66 | 0.00 | 1.53 | 0.02 | 0.47 | 0.54 | 0.10 | 0.02 | 0.00 |

**Notes:** Genera are arranged in decreasing order. For sample codes, see Table 1. M_tot_ = mean relative abundance averaged over all samples; M_Cil_ = mean relative abundance averaged over hindgut ciliate samples; M_Int_ = mean relative abundance averaged over all ciliate-free hindgut samples.
